# A Hybrid Multi-Strategy Differential Creative Search Optimization Algorithm and Its Applications

**DOI:** 10.3390/biomimetics10060356

**Published:** 2025-06-01

**Authors:** Yuanyuan Zhang, Longquan Yong, Yijia Chen, Jintao Yang, Mengnan Zhang

**Affiliations:** School of Mathematics and Computer Science, Shaanxi University of Technology, Hanzhong 723001, China

**Keywords:** differentiated creative search algorithm, refined set, clustering process, double Q-learning, mechanical optimization

## Abstract

To address the issues of uneven initial distribution and limited search accuracy with the traditional divergent quantum-inspired differential search (DCS) algorithm, a hybrid multi-strategy variant, termed DQDCS, is proposed. This improved version overcomes these limitations by integrating the refined set strategy and clustering process for population initialization, along with the double Q-learning model to balance exploration and exploitation This enhanced version replaces the conventional pseudo-random initialization with a refined set generated through a clustering process, thereby significantly improving population diversity. A novel position update mechanism is introduced based on the original equation, enabling individuals to effectively escape from local optima during the iteration process. Additionally, the table reinforcement learning model (double Q-learning model) is integrated into the original algorithm to balance the probabilities between exploration and exploitation, thereby accelerating the convergence towards the global optimum. The effectiveness of each enhancement is validated through ablation studies, and the Wilcoxon rank-sum test is employed to assess the statistical significance of performance differences between DQDCS and other classical algorithms. Benchmark simulations are conducted using the CEC2019 and CEC2022 test functions, as well as two well-known constrained engineering design problems. The comparison includes both recent state-of-the-art algorithms and improved optimization methods. Simulation results demonstrate that the incorporation of the refined set and clustering process, along with the table reinforcement learning model (double Q-learning model) mechanism, leads to superior convergence speed and higher optimization precision.

## 1. Introduction

Optimization theory and its practical applications are undergoing unprecedented transformation in contemporary industrial practice, with their core value lying in the targeted enhancement of key system performance indicators. Driven by the continuous expansion of engineering frontiers and the rapid advancements in computational science, classical optimization paradigms—such as Newton’s method and gradient descent—have increasingly revealed their limitations [1,2,3]. Applied to modern engineering problems with high dimensionality and strong nonlinearity, these methods often face exponential time complexity, making exact solutions practically infeasible. This practical challenge has catalyzed the vigorous development of metaheuristic optimization techniques. These algorithms, inspired by natural phenomena or social behavior [4,5], offer promising alternatives by delivering global or high-accuracy approximate solutions under acceptable computational costs. The balance between solution quality and computational efficiency makes modern metaheuristic algorithms—such as genetic algorithms (GA) [6], particle swarm optimization (PSO) [7], and ant colony optimization (ACO) [8]—powerful tools with wide-ranging applicability. These methods have demonstrated exceptional performance in solving complex optimization problems across various interdisciplinary domains, including intelligent manufacturing, logistics scheduling, and financial modeling [9,10,11].

As a vital branch of metaheuristic algorithms, swarm intelligence optimization techniques have attracted increasing attention in the academic community in recent years. These algorithms emulate the cooperative behavior of biological populations—such as the flock migration model in particle swarm optimization (PSO), the mating behavior pattern in butterfly optimization algorithm (BOA) [12], and the deep-sea migration strategy in the salp swarm algorithm (SALP) [13]—demonstrating distinctive computational advantages. Their simple and intuitive structural design, rapid convergence, and multimodal optimization capabilities enable excellent adaptability in solving complex, nonlinear, and high-dimensional engineering optimization problems. Notably, bio-inspired optimization paradigms are undergoing continuous innovation. Modern engineering design methodologies have accelerated the iterative process through parallel search mechanisms and have adopted dynamic balance strategies to coordinate exploration and exploitation. These approaches have delivered significant application value in improving mechanical system performance, reducing operational costs, and optimizing resource allocation efficiency. For instance, Houssein et al. [14] proposed a dimension learning-enhanced equilibrium optimizer that constructs multi-level feature interaction mechanisms, significantly improving the lesion segmentation accuracy of COVID-19 lung CT images, particularly in low-contrast regions. Alkayem et al. [15] developed an adaptive pseudo-inverse stochastic fractal search algorithm that employs intelligent dimensionality reduction strategies within the solution space, enabling robust detection of subtle defects in complex steel structure damage assessments and greatly enhancing diagnostic reliability. Abdollahzadeh et al. [16] introduced the puma optimization (PO) algorithm, which simulates jaguar-inspired intelligent behavior for designing exploration–exploitation mechanisms. By integrating a hyper-heuristic phase transition strategy, the algorithm adaptively balances optimization stages according to problem characteristics, thereby substantially improving the capability to solve complex optimization problems. Liu et al. [17] integrated the Q-learning mechanism from reinforcement learning to dynamically hybridize the Aquila optimizer (AO) with the improved arithmetic optimization algorithm (IAOA). By reconstructing the mathematical acceleration function, they achieved a better balance between global search and local exploitation while optimizing the reward model to enhance decision-making efficiency. Wang et al. [18] proposed an underwater image enhancement method based on reinforcement learning. The reward was constructed by a human visual perception index, the features were extracted by residual network, and the image was gradually optimized by combining various enhancement algorithms. The visual effect could be improved without reference to the image. Li et al. [19] proposed a joint detection and tracking (IDT) method based on reinforcement learning, which guided the detector to dynamically optimize the threshold through the feedback prediction information of the tracker, and established an adaptive detection gate in the range-Doppler diagram, thus effectively improving the detection probability and tracking continuity of high-frequency ground wave radar for weak targets. Zhu et al. [20] combined the global exploration capability of the black-winged kite algorithm (KA), the local optimization strength of particle swarm optimization (PSO), and the mutation strategy of differential evolution (DE) to balance search abilities, effectively preventing premature convergence and significantly improving both the convergence speed and solution accuracy in high-dimensional optimization problems. However, with the increasing complexity of engineering optimization tasks—characterized by multi-constraint coupling and high-dimensional nonlinearity—current swarm intelligence algorithms have begun to exhibit theoretical limitations when handling non-convex solution spaces and adapting to dynamic environments. In particular, for complex system designs involving strong time-varying properties and multi-objective conflicts, challenges arise in ensuring convergence stability and maintaining well-distributed solution sets.

The differential creative search (DCS) algorithm [21] represents a cutting-edge advancement in the field of metaheuristic optimization. This algorithm introduces an innovative multi-stage co-evolution framework in which a heterogeneous knowledge transfer mechanism dynamically integrates population experience, while a dynamic solution space reconstruction strategy enhances adaptability to complex optimization problems. DCS demonstrates strong global convergence performance in tackling high-dimensional, non-convex, and multimodal engineering optimization tasks. Liu et al. [22] proposed a hybrid approach incorporating an opposition-based learning strategy along with an adaptive reset mechanism that balances fitness and distance. This design encourages low-performance individuals to migrate toward the vicinity of the optimal solution, thereby facilitating the exploration of promising regions in the search space. Cai et al. [23] improved the shortcomings of DCS algorithm through collaborative development mechanism and population evaluation strategy. However, DCS still suffers from limitations in terms of solution distribution bias and insufficient local convergence precision during iterations, which restrict its ability to effectively explore the boundary regions of the search space and identify the global optimum. To address these challenges, we propose an improved initialization strategy that integrates a refined set with a clustering process to enhance the diversity of the initial population. Furthermore, we incorporate a double Q-learning strategy using dual Q-tables, enabling a more effective balance between exploration and exploitation. This balance empowers the algorithm to explore unknown regions in complex environments more effectively, thereby reducing the risk of premature convergence to local optima.

The main contributions of the proposed algorithm in this study are summarized as follows.

(1) A novel algorithm is developed by integrating a refined set, a clustering process, and a double Q-learning strategy into the differential creative search (DCS) framework. Ablation studies are conducted to verify that each of these strategies contributes positively to the performance enhancement of the DCS algorithm.
(2) The proposed DQDCS algorithm is benchmarked against ten state-of-the-art algorithms on the CEC2019 and CEC2022 test suites. Extensive simulations demonstrate the superior performance of DQDCS. Its improvements are visualized through convergence curves and boxplots, and further validated by the Wilcoxon rank-sum test to confirm its overall effectiveness.
(3) The DQDCS algorithm is applied to two real-world constrained engineering design problems: the design of hydrostatic thrust bearings and the synchronous optimal pulse width modulation (SOPWM) problem in three-level inverters. In both cases, the goal is to minimize the objective function under complex constraints. The results show that DQDCS is particularly suitable for solving practical engineering optimization problems.

The remainder of this study is organized as follows. Section 2 introduces the fundamentals of the differential creative search (DCS) algorithm. Section 3 elaborates on the three enhancement strategies incorporated into DQDCS. Section 4 reports the experimental comparisons between DQDCS and other algorithms on the CEC2019 and CEC2022 benchmarks. Section 5 presents the application of DQDCS to practical engineering problems and offers a comprehensive analysis of the results. Finally, Section 6 concludes the study.

## 2. Differentiated Creative Search Algorithm Thoughts and Process

The differentiated creative search (DCS) algorithm is a swarm intelligence-based optimization method whose core framework integrates differentiated knowledge acquisition (DKA) and creative realism (CR). A dual-strategy mechanism is employed to balance divergent and convergent thinking. In this framework, high-performing individuals adopt a divergent thinking strategy, utilizing existing knowledge and creative reasoning to conduct global exploration guided by the Linnik distribution. In contrast, the remaining individuals adopt a convergent thinking strategy, integrating feedback from both the team leader and peer members to perform local exploitation. This process involves local optimization informed by both elite and randomly selected individuals. The overall procedure of the DCS algorithm is summarized as follows.

### 2.1. Initialization

The initial population X is randomly generated, with each individual represented as a random solution:

$X_{i}=[x_{i,1},x_{i,2},x_{i,3},\ldots ,x_{i,D}]$ satisfying Equation (1):

(1) $$x_{i,d}=L+U(0,1)\times (U-L)$$

where U and L represent the lower and upper bounds of the *d*-th dimension, respectively, and U(0,1) follows a uniform distribution on the interval (0,1).

### 2.2. Differentiated Knowledge Acquisition

Differentiated knowledge acquisition (DKA) emphasizes the rate at which new knowledge is acquired, exerting differential effects on individual agents. These effects are primarily manifested through the modification of the individual’s existing knowledge attributes or dimensional components. The parameter $\eta_{i,t}$ denotes the quantified knowledge acquisition rate of the *i*-th individual at iteration *t*, as defined in Equation (2).

(2) $$\eta_{i,t}=\left\{\begin{array}{l} \frac{1}{2}\times \left(\left[U\left(0,1\right)\times \varphi_{i,t}\right]+1\right),\;if\;U\left(0,1\right)\leq \varphi_{i,t}\\ \frac{1}{2}\times \left(\left[U\left(0,1\right)\times \varphi_{i,t}\right]+0\right),\;\mathrm{otherwise} \end{array}\right.$$

In Equation (2), $\varphi_{i,t}$ represents the coefficient associated with variable φ for the individual at the t-th iteration, and is calculated using Equation (3). Here, *NP* denotes the population size, and $R_{i,t}$ indicates the rank of the *i*-th individual at the beginning of iteration t. The influence of the DKA process on each component of $X_{i}$ is described in Equation (4), where $j_{rand}∼U\left(\left\{1,2,\ldots ,D\right\}\right)$ denotes an integer uniformly selected from the set $\left\{1,2,\ldots ,D\right\}$ and D represents the dimensionality of the problem.

(3) $$\varphi_{i,t}=0.25+0.55\times \sqrt{R_{i,t}/NP}$$

(4) $$\left\{\begin{array}{l} j_{rand}∼U\left(\left\{1,2,\ldots ,D\right\}\right)\\ v_{i,d}=\left\{\begin{array}{l} v_{i,d},\;if\;U\left(0.1\right)\leq \eta_{i,t}\;or\;d=j_{rand}\\ x_{i,d},\;otherwise \end{array}\right. \end{array}\right.$$

### 2.3. Convergent Thinking

The strategy for low-performing individuals leverages the knowledge base of high performers and incorporates the stochastic contributions of two randomly selected team members into the solution proposed by the current individual. The inertia weight *F* is generated randomly, and this process is described by Equation (5), where *F* the inertia weight and $X_{best}$ represents the best global solution in the current population.

(5) $$V_{i}=F\times X_{best}-\left(F\times X_{r2}-X_{i}\right)+F\times \left(X_{r1}-X_{i}\right)$$

$X_{i}$ draws upon convergent thinking by integrating the information provided by team members $X_{r1}$ and $X_{r2}$, thereby refining the knowledge of the team leader, $X_{best}$, as described by Equation (6). Here, the coefficient $\lambda_{t}$ governs the extent to which peer influence shapes an individual’s social cognition within the team environment. It reflects the degree to which team dynamics affect individual perspectives. The value of $\lambda_{t}$ decreases over time, as defined in Equation (7), where *NFE_T_* and *NFE*_max_ denote the number of function evaluations in the current iteration and the maximum allowable number of function evaluations, respectively.

(6) $$V_{i}=\omega \times X_{\mathrm{best}}+\lambda_{t}\times \left(X_{r2}-X_{i}\right)+\omega_{i,t}\times \left(X_{r1}-X_{i}\right)$$

(7) $$\lambda_{t}=0.1\times 0.518\times (1-\sqrt{NFE_{T}/NFE_{\max}})$$

Two random individuals, $x_{r1}$ and $x_{r2}$, are selected, and a new candidate solution is generated by incorporating the best individual with stochastic components, as defined in Equation (8).

(8) $$v_{i,d}=\omega \cdot x_{\mathrm{best},d}+\lambda_{t}\cdot (x_{r2,d}-x_{i,d})+\omega_{i,t}\cdot (x_{r1,d}-x_{i,d})$$

### 2.4. Divergent Thinking

A random individual $x_{r1}$ is selected, and a new candidate solution is generated using the Linnik distribution, as defined in Equation (9), where LK(α,σ) denotes a random variable drawn from the Linnik distribution with parameters α=0.618 and σ=0.05.

(9) $$v_{i,d}=x_{r1}+LK(\alpha ,\sigma )$$

### 2.5. Team Diversification

As the team continues to evolve, it generates increasingly diverse ideas. To maintain diversity and adaptability, the DCS algorithm replaces underperforming members with newly generated individuals. The equation used to generate new individuals is provided in Equation (10).

(10) $$V_{NP}=L+U(0,1)\times (U-L)$$

### 2.6. Offspring Population

For each individual $X_{i,t}$, a trial solution $V_{i,t}$ is generated. The decision to retain or replace the original solution is based on a comparison of their fitness values. If the trial solution $V_{i,t}$ exhibits superior fitness, it replaces the original solution $X_{i,t}$; otherwise, the original solution is retained. This process is formulated in Equation (11).

(11) $$X_{i,t+1}=\left\{\begin{array}{l} V_{i,t},\;if\;f(V_{i,t})\;\leq \;f(X_{i,t})\\ X_{i,t},\;\mathrm{otherwise} \end{array}\right.$$

In each iteration, all individuals $X_{i,t+1}$ in the newly generated population are evaluated, and the best global solution $X_{\mathrm{best},t}$ is subsequently updated. This process is formally defined in Equation (12).

(12) $$X_{\mathrm{best},t+1}=\left\{\begin{array}{l} X_{i,t+1},\;if\;f(X_{i,t+1})<f(X_{\mathrm{best},t})\\ X_{\mathrm{best},t},\;\mathrm{otherwise} \end{array}\right.$$

Algorithm 1 outlines the detailed steps of the DCS algorithm described above.
**Algorithm 1:** Particle swarm optimizationStep 1: Random Initialization of the Population The initial population is generated randomly to ensure diversity in the solution space. Step 2: Fitness Evaluation The fitness of each individual is evaluated by computing its objective function value, which reflects the individual’s performance on the optimization problem. Step 3: Determination of the Number of High-Performance Individuals The number of top-performing individuals is determined based on a predefined proportion of the population. Step 4: Initialization of Iteration Counter and Parameters Set the iteration counter t = 1, the number of function evaluations *NFE* = 0, and the probability of population migration to 0.5. Step 5: Main Optimization Loop Continue the optimization process while *NFE* < *NFE*_max_, repeating the position updating and fitness evaluation steps.

## 3. Hybrid Multi-Strategy Differentiated Creative Search Algorithm

Firstly, the DCS algorithm initializes the population using pseudo-random numbers, which results in limited population diversity and a lack of target-oriented search in the early stages, thereby reducing optimization efficiency. Secondly, the algorithm relies heavily on the best-performing individual during position updates, making it susceptible to premature convergence and hindering its ability to escape local optima. These issues ultimately lead to reduced optimization accuracy and a slower convergence rate.

To address these limitations, targeted strategies are introduced to enhance the algorithm’s overall performance. Previous studies have shown that the diversity of the initial population significantly influences the algorithm’s ability to converge rapidly and accurately. A higher degree of initial diversity allows the algorithm to explore a broader solution space during the search process, thereby increasing the likelihood of identifying the global optimum.

To improve population diversity, generate higher-quality initial solutions, and effectively overcome the limitations of the basic DCS algorithm, a refined set strategy and clustering strategy are incorporated during the population initialization phase. Although the DCS algorithm enhances global exploration through its “creativity” mechanism, it lacks sufficient flexibility in dynamic environments, such as path planning with moving obstacles for UAVs (unmanned aerial vehicles) or real-time demand changes in industrial scheduling.

The incorporation of double Q-learning allows the algorithm to interact continuously with the environment, facilitating real-time perception and autonomous decision-making. This enables the DCS algorithm to adapt its search strategy more precisely under dynamic conditions, thereby maintaining high operational efficiency. Furthermore, the learning capability of double Q-learning enhances the algorithm’s generalization ability, enabling robust performance in unseen scenarios and increasing the practical value of intelligent optimization techniques.

### 3.1. Refined Set Initialization

Compared to traditional pseudo-random initialization, the refined set strategy achieves a more uniform distribution of the population across the search space through a carefully designed sampling method. This uniformity reduces the likelihood of the algorithm becoming trapped in local optima during the early stages of optimization. By promoting a broader distribution of individuals, the algorithm gains increased opportunities to explore diverse regions and identify superior solutions.

The mathematical formulation of the refined set strategy is presented in Equations (13) and (14), where *L* and *U* represent the lower and upper bounds of the *d*-th dimension, respectively, and i indicates the index of the *i*-th individual. The parameter p denotes the smallest prime number that satisfies specific conditions, and $\mathrm{mod}\left(d\times i,p\right)$ refers to the result of the modulo operation applied to d×i with respect to *p*. Specifically, it involves calculating the remainder of d×i divided by *p*. The value of *p* directly influences the initial distribution of the population. A larger *p* value enhances global exploration capability by promoting a more uniform distribution, while a smaller *p* value may cause the algorithm to prematurely enter the local exploitation phase. The parameter *p* is calculated using Equation (15), where *N* represents the set of natural numbers and next_prime(d×i) denotes the smallest prime number greater than d×i .

(13) $$M=\frac{\mathrm{mod}\left(d\times i,p\right)}{p}$$

(14) $$x_{i,d}=L+M\times (U-L)$$

(15) $$p=\left\{\begin{array}{l} d\times i\;\;\;\;\;\;d\times i\in N\\ next\_prime(d\times i)\;d\times i\notin N \end{array}\right.$$

### 3.2. Clustering Process

After the population is generated using the refined set strategy, a clustering algorithm is applied for further enhancement. In this study, the k-means clustering method is adopted to allow the DCS algorithm to focus on salient features during the learning phase while minimizing the impact of low-quality data. This process improves the algorithm’s generalization ability on unseen data, thereby increasing its robustness and stability in practical applications. The detailed implementation steps are presented as follows.

(1) Select the Cluster Centers

Randomly select *k* points as the initial cluster centers $C_{1},C_{2},\ldots ,C_{k},$ where $C_{j}=\left(c_{j,1},c_{j,2},\ldots ,c_{j,D}\right)$. In this experiment, the value of *k* is determined by Equation (16), where NP denotes the population size. The value of *k* linearly varies with the population size, ensuring that each cluster contains a sufficient number of individuals. When the population is large, increasing the value of *k* allows for capturing more intricate details. Conversely, when the population is small, reducing the value of *k* helps prevent excessive subdivision.

(16) $$k=\left\lceil \frac{NP}{5}\right\rceil$$

(2) Calculate the distance and assign individuals

For each individual $x_{i}$ in the population, the Euclidean distance between $x_{i}$ and each cluster center $C_{j}$ is calculated. The individual $x_{i}$ is then assigned to the cluster corresponding to the nearest cluster center. This is specifically expressed by Equation (17).

(17) $$d\left(x_{i},C_{j}\right)=\sqrt{\sum_{d=1}^{D}{\left(x_{i,d}-c_{j,d}\right)}^{2}}$$

(3) Update the cluster centers

For each cluster, the cluster center is recalculated. Let the set of individuals in the *j*-th cluster be denoted $S_{j}$. The new cluster center $C_{j}^{\prime }=\frac{1}{\left|S_{j}\right|}\sum_{x_{i}\in S_{j}}x_{i}$ is computed as the mean of the individuals in $S_{j}$, where $\left|S_{j}\right|$ denotes the number of individuals in the set $S_{j}$.

(4) Iterative process

Repeat steps 2 and 3 until the cluster centers no longer undergo significant changes.

(5) Density-based uniform selection

Calculate the local density of each individual $x_{i}\in S_{j}$, denoted $\rho_{i}=\frac{1}{\left|S_{j}\right|}\sum_{x_{k}\in S_{j}}\exp\left(-{\left(\frac{d\left(x_{i},x_{k}\right)}{d_{cut}}\right)}^{2}\right)$, where $d_{cut}$ is the truncation distance used in the density calculation. Individuals with moderate density are selected, and the final initialized population is given by Equation (18).

(18) $$X_{\mathrm{final}}=U_{j=1}^{k}Selected_{j}$$

### 3.3. Double Q-Table Reinforcement Learning Model (Double Q-Learning Model)

The Q-learning model consists of five fundamental components: the agent, environment, action, state, and reward. Its operational procedure can be succinctly described as a cyclical interaction of state transition, action selection mechanism, and reward feedback. In traditional Q-learning, a single Q-table is employed to store the estimated values of state–action pairs. However, this approach often suffers from issues such as overestimation bias and limited exploration capacity. To mitigate these limitations, double Q-learning introduces two independent Q-tables, each of which is updated independently. This dual-table framework significantly reduces the estimation bias inherent in the single-table approach and enables a more balanced, robust evaluation of the learned policy.

Let $S=\left\{s_{1},s_{2},\ldots ,s_{m}\right\}$ denote the set of environment states and $A=\left\{a_{1},a_{2},\ldots ,a_{n}\right\}$ represent the set of actions that the agent can execute. In each iteration, the agent occupies a certain state s∈S and selects an action a∈A to perform. After executing the action, the environment provides a reward $r_{t+1}$ and a new state $s_{t+1}$. The reward is computed according to Equation (19), where $X_{\mathrm{new}}$ denotes the solution at the new position and $X_{i,1}$ represents the solution at the current position. Upon receiving this information, the agent evaluates the expected value $Q\left(s_{t},a_{t}\right)$ for each possible action.

**Figure 1:**
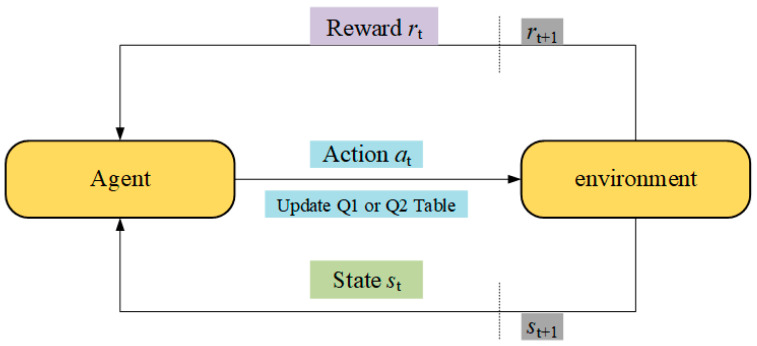
Operation process of double Q-learning.

In double Q-learning, either $Q_{1}$ or $Q_{2}$ is selected for updating with equal probability. The update equations are provided in Equations (20) and (21). In the equations, $s_{t}$ represents the state of the agent, $a_{t}$ denotes the action executed by the agent, $r_{t+1}$ is the immediate reward obtained by the agent after executing the action, and γ is the discount factor, which penalizes future rewards. When γ=0, double Q-learning considers only the current reward; when γ=1, it prioritizes long-term rewards, α is the learning rate, typically within the interval $\left[0,1\right]$. Double Q-learning maintains two Q-tables, traditional Q-learning, but with distinct update logic. Figure 1 simplified illustration example of the operational process of double Q-learning, the arrows in the figure indicate the flow direction of the data, while the dotted line represents the dividing line of time, the transition from time t to t+1. The agent alternates between updating the two Q-tables and combines information from both when selecting actions, thereby reducing estimation bias and enhancing policy robustness.

(19) $$r_{t+1}=f(X_{\mathrm{new}})-f(X_{i,1})$$

(20) $$Q_{1}\left(s_{t},a_{t}\right)=Q_{1}\left(s_{t},a_{t}\right)+\alpha \left[r_{t+1}+\gamma Q_{2}\left(s_{t+1},\underset{a}{\arg\max}Q_{1}\left(s_{t+1},a\right)\right)-Q_{1}\left(s_{t},a_{t}\right)\right]$$

(21) $$Q_{2}\left(s_{t},a_{t}\right)=Q_{2}\left(s_{t},a_{t}\right)+\alpha \left[r_{t+1}+\gamma Q_{1}\left(s_{t+1},\underset{a}{\arg\max}Q_{2}\left(s_{t+1},a\right)\right)-Q_{2}\left(s_{t},a_{t}\right)\right]$$

In the DCS algorithm, the last individual is considered inefficient and updated using random initialization, which often results in low-quality solutions. To address this issue, double Q-learning introduces two independent Q-tables and employs a dual Q-table mechanism to improve solution quality. This separation makes the calculation of target Q-values more reliable, helping to reduce estimation bias.

### 3.4. Ablation Experiment

To evaluate the effectiveness of the proposed strategies, four representative test functions from the CEC2019 benchmark suite were selected. The performance of the refined set strategy, the clustering process strategy, their combination, and the double Q-learning strategy was systematically compared. Each algorithm was executed for 500 iterations across 30 independent runs. Specifically, D1 adopts the refined set strategy, D2 employs the clustering process strategy, D3 integrates both the refined set and clustering process strategies, and D4 incorporates the double Q-learning mechanism. The corresponding results are summarized in Table 1. Experimental results demonstrate that population initialization using the combined refined set and clustering process significantly enhances the algorithm’s capability to approach the global optimum. Moreover, the D4 variant, augmented with the double Q-learning strategy, achieves the best overall fitness across all test functions.

### 3.5. Hybrid Multi-Strategy DQDCS Algorithm

The DQDCS algorithm integrates both the refined-point set strategy and a clustering-based approach, and further incorporates a double Q-learning model to construct a multi-strategy hybrid optimization framework. The refined-point set is generated through mathematically guided sampling techniques to ensure a more uniform distribution of individuals across the search space, thereby replacing conventional pseudo-random initialization methods. Such a distribution promotes broader coverage of the solution space and mitigates early-stage search blind spots. A high-quality initial population enhances the algorithm’s ability to converge more reliably toward optimal solutions and reduces performance fluctuations caused by poor initial positioning.

In contrast to purely random initialization, the refined-point set strategy employs structured sampling to diminish the randomness-induced variability in the initial population, thereby improving the algorithm’s robustness. Moreover, this strategy can be tailored to the specific characteristics of the optimization problem; for example, in constrained optimization scenarios, it helps ensure that the initial population satisfies constraint conditions, thus avoiding infeasible solutions and enhancing overall algorithmic stability.

The clustering strategy divides the population into multiple subgroups, with each subgroup representing distinct regions or features of the search space. This structural partitioning aids in preserving diverse solution patterns throughout the optimization process, thereby reducing the risk of premature convergence to local optima. By maintaining diversity and promoting exploration, the algorithm is better positioned to locate the global optimum efficiently.

**Figure 2:**
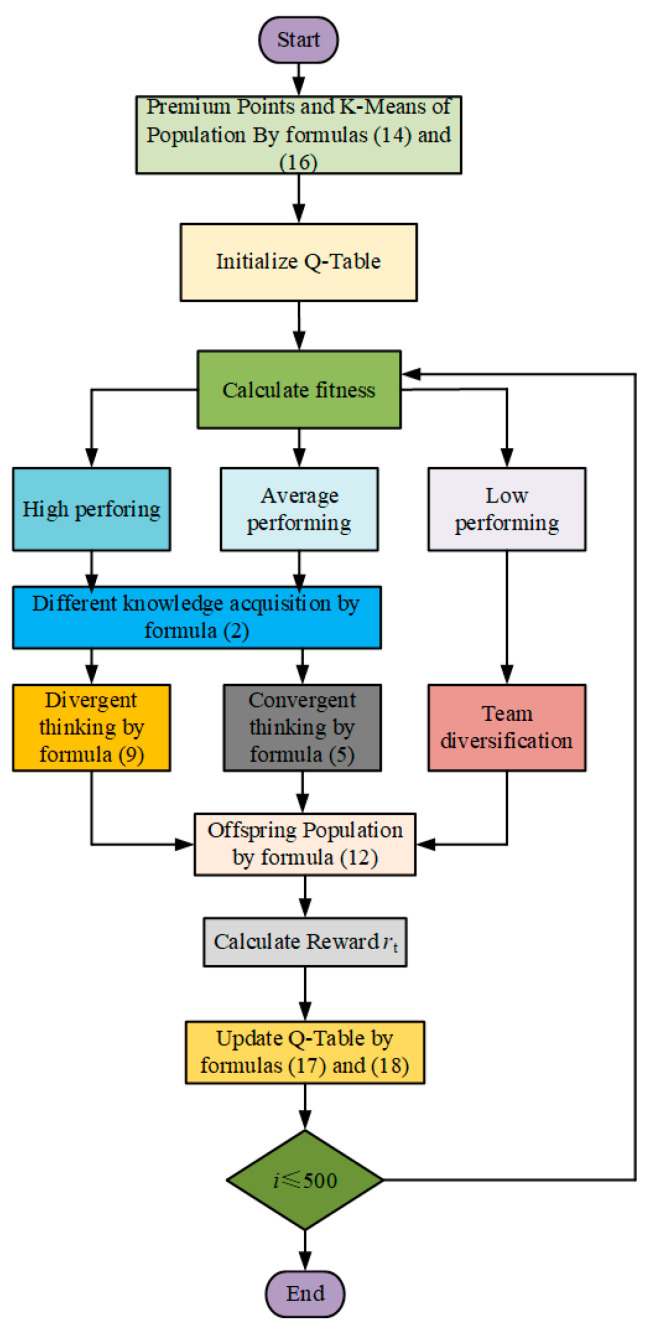
DQDCS flowchart.

By integrating the probability distributions and maximum values derived from two independent Q-tables, the double Q-learning mechanism enables more balanced action selection between exploration and exploitation. Within the DQDCS algorithm, this dual-Q-table framework effectively mitigates the estimation bias commonly associated with single-Q-table implementations and facilitates more comprehensive policy evaluation, thereby enhancing the algorithm’s global search capability. Specifically, double Q-learning selects the optimal action based on the current state, which subsequently guides population updates. This approach allows for broader exploration during the early stages of optimization while gradually shifting toward refined exploitation of promising regions in the later stages. As such, the algorithm achieves accelerated convergence without compromising solution quality. A flowchart of the DQDCS algorithm is illustrated in Figure 2.

Algorithm 2 presents the pseudocode of the proposed DQDCS algorithm.
**Algorithm 2:** DQDCS algorithm.Initialize the population using Equation (1); Evaluate fitness for all individuals; Determine the refined set via the clustering process; Initialize Q-tables to zero; Set key parameters: exploration threshold pc, golden ratio, η and φ values; while the number of function evaluations (nfe) < max_nfe do  Sort the population by fitness;  Identify the best solution *x*_best;  Compute λ_t_ using Equation (7);  for each individual i do    Compute η_i_ and φ_i_ using Equations (2) and (3);    Determine behavior category (high-, average-, or low-performing);    if i is low-performing and rand < pc then      Generate a new solution randomly;    else if i is high-performing then      Select r_1_ ≠ i;      Update selected dimensions using Equation (8);    else//average-performing      Select r_1_, r_2_ ≠ i;      Compute ω_i_;      Update selected dimensions using Equation (8);    end if    Apply reflection-based boundary handling;    Evaluate fitness of the new solution;    If improved, update position and fitness;    Compute reward from fitness change;    Update Q_1_ using Q_2_ for value estimation (Equation (20));    Update Q_2_ using Q_1_ similarly (Equation (21));  end for  Update the best solution and record convergence data; end while Return best solution, best fitness;

### 3.6. Complexity Analysis of the Algorithm

The implementation of the DQDCS algorithm involves certain design challenges, yet it maintains relatively low computational complexity. The overall complexity is stage-dependent, as each phase—initialization, fitness evaluation, and solution generation—contributes differently to the total computational cost.

In general, the DQDCS algorithm comprises three fundamental procedures: population initialization, fitness evaluation, and generation of new solutions. The main loop iterates for a maximum of Max_iter iterations, and in each iteration, operations are performed for each of the N search agents.

The computational complexity of the initialization phase is O(N), owing to the refined set-based initialization method and clustering process. Additionally, the time complexity for fitness evaluation depends on the complexity of the objective function, denoted O(F). Therefore, the overall computational complexity of the algorithm can be expressed as O(Max_iter × N × F).

## 4. Simulation Environment and Result Analysis

In the field of optimization, particularly in the study of evolutionary algorithms and metaheuristic methods, validating the effectiveness of proposed algorithms is of paramount importance, as these approaches are expected to address complex challenges encountered in real-world applications. To assess their performance, standardized test cases or well-established benchmark problems are commonly employed. These benchmark evaluations offer a unified platform for objective comparison, enabling fair and consistent performance assessment across different algorithms and facilitating a rigorous analysis of their strengths and limitations.

This experiment was conducted using MATLAB 2023 and a Windows 11 operating system with Intel Core i5-13400 CPU and 8 GB RAM (Intel Corporation, Santa Clara, CA, USA). The CEC2019 [24] and CEC2022 [25] benchmark functions were employed to evaluate the performance of the DQDCS algorithm. These benchmark functions enable a systematic comparison between DQDCS and other state-of-the-art metaheuristic algorithms, thereby verifying the competitiveness and applicability of the proposed method in solving complex optimization problems. This evaluation framework ensures scientific rigor and provides clear directions for further algorithmic enhancements.

Considering the inherent stochastic nature of metaheuristic algorithms, relying on a single run for each benchmark function may lead to unreliable conclusions. Therefore, multiple simulations were conducted for each algorithm, including puma algorithm [16], the original differentiated creative search algorithm (DCS) [21], the multi-strategy hybrid DQDCS algorithm, a differentiated creative search algorithm with multi-strategy improvement (MSDCS) [22], Chernobyl disaster optimizer (CDO) [26], adaptive spiral flying sparrow search algorithm (ASFSSA) [27], waterwheel plant algorithm (WWAP) [28], subpopulation improved grey wolf optimizer with Gaussian mutation and Lévy flight (SPGWO) [29], dung beetle optimizer (DBO) [30] and nonlinear randomly reuse-based mutated whale optimization algorithm (NRRMWOA) [31]. The detailed experimental results are presented as follows.

### 4.1. The CEC2019 Benchmark Functions Are Employed for Performance Evaluation

The CEC2019 benchmark functions are specifically designed to evaluate and compare the performance of optimization algorithms. They encompass a diverse set of challenging optimization problems, including multimodal, high-dimensional, and dynamic characteristics, thereby closely simulating real-world complexities. Given the inherent stochastic nature of metaheuristic algorithms, a single run per benchmark function is insufficient to reliably demonstrate an algorithm’s effectiveness. To enhance the reliability and fairness of performance evaluation, each algorithm was independently tested 100 times on each benchmark function, with a maximum of 500 iterations per run. The population size was consistently established at 200 individuals.

#### 4.1.1. CEC2019 Optimization Accuracy Analysis

To accurately evaluate and compare the performance on the CEC2019 benchmark functions, the experimental results are summarized in terms of the best, mean, and standard deviation (Std) values. In the result tables, the best mean values, which serve as key performance indicators, are highlighted with underlining. The detailed outcomes are presented in Table 2.

As shown in the statistical results in Table 2, the proposed DQDCS algorithm consistently achieves the best values on all functions from F1 to F10. Moreover, it obtains the best mean performance on F3–F10 compared to other competing algorithms. In addition, the DQDCS achieves the smallest standard deviation on functions F3, F5, F6, F8, and F9, demonstrating superior robustness. These findings indicate that DQDCS not only offers stable performance but also exhibits highly competitive exploration capability among the compared algorithms.

#### 4.1.2. CEC2019 Convergence Curve Analysis

**Figure 3:**
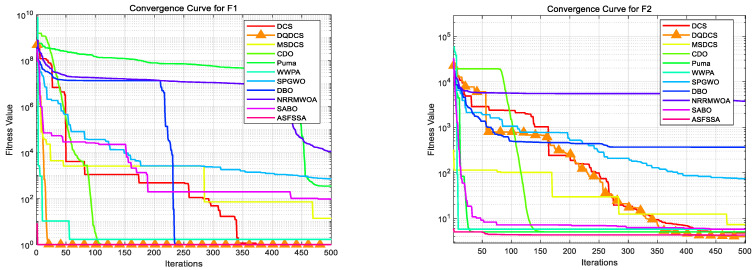

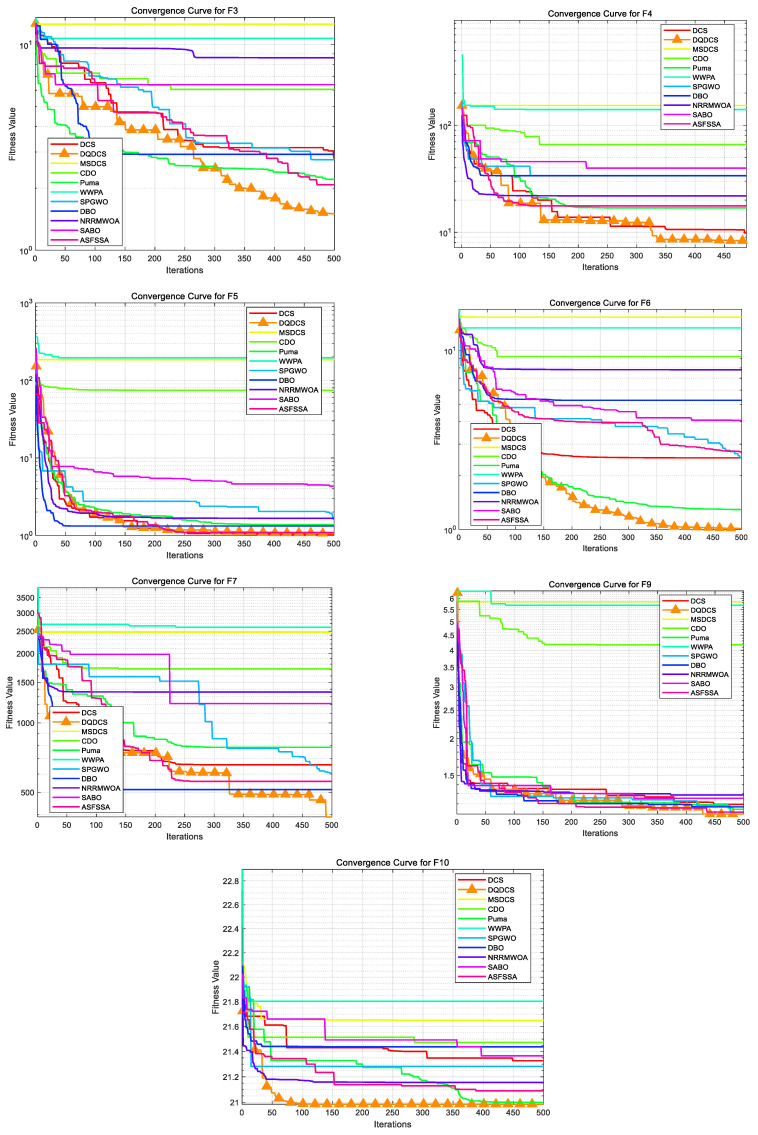
Convergence curve of CEC2019 benchmark tests.

To facilitate a more intuitive comparison of the convergence behavior across different functions in the CEC2019 benchmark functions, convergence curves for the DQDCS algorithm and ten other competing algorithms were plotted. As shown in Figure 3, the horizontal axis represents the number of iterations, while the vertical axis denotes the fitness value. It can be observed that the DQDCS algorithm demonstrates superior convergence accuracy on functions F1–F7, F9, and F10. Notably, it is capable of approaching the optimal value at the early stages of the search process, particularly on functions F1 and F10, where the curves converge almost linearly to the optimum. These results indicate that the DQDCS algorithm exhibits relatively strong performance and that the incorporation of multi-strategy enhancements is both feasible and effective in improving optimization precision.

#### 4.1.3. CEC2019 Boxplot Analysis

**Figure 4:**
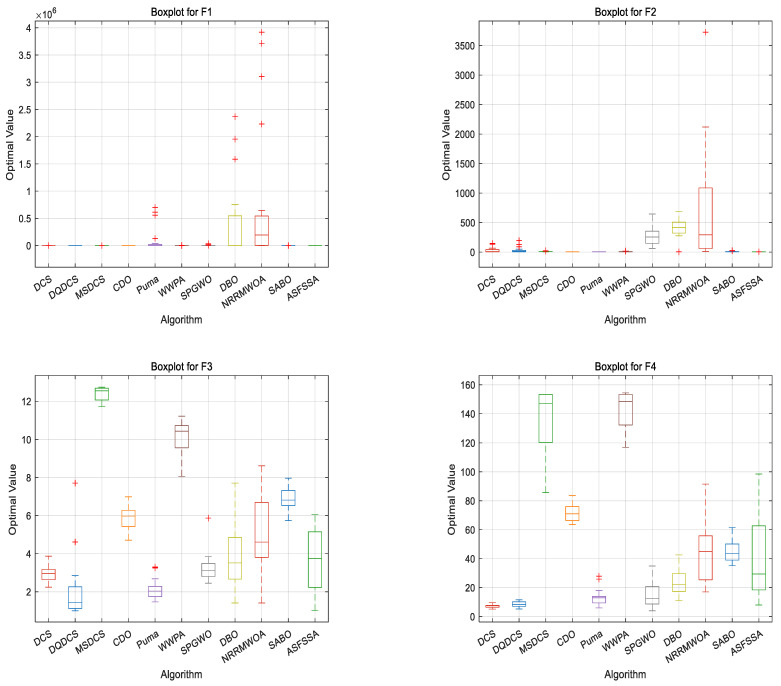

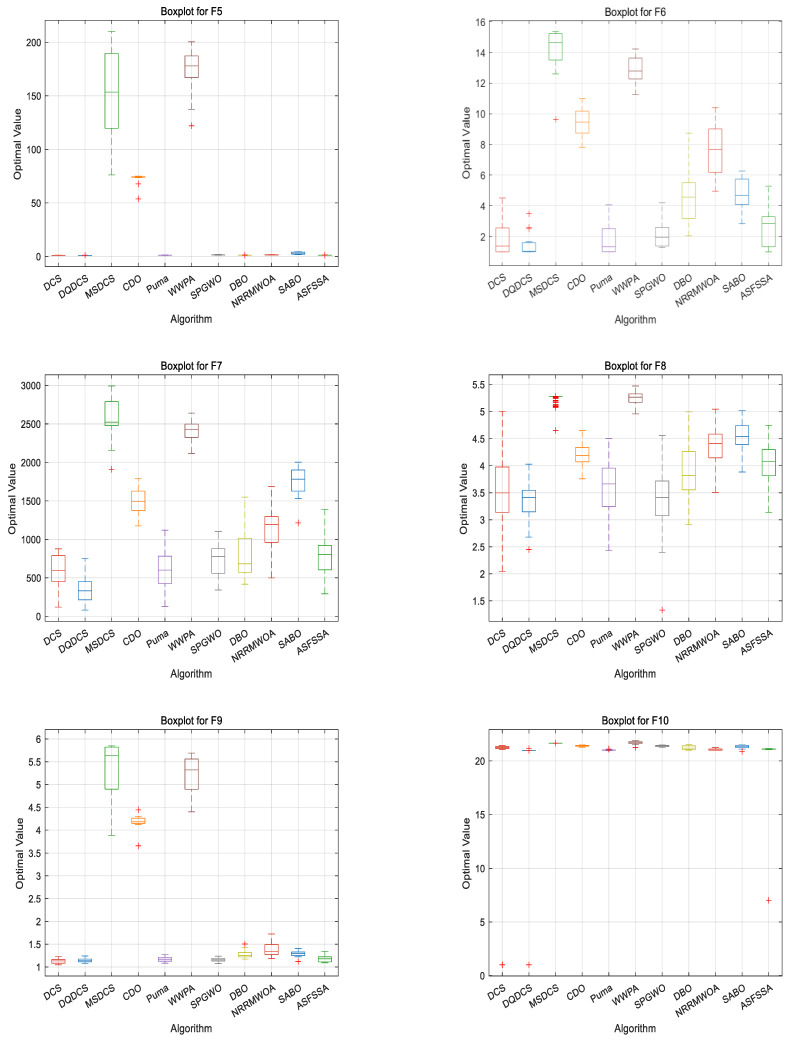
Boxplots of CEC2019 benchmark functions. “+” is the abnormal data in the box plot.

To compare the performance of different algorithms across multiple runs—particularly in terms of stability and robustness—boxplots are employed as an evaluation tool. As shown in Figure 4, for the DQDCS algorithm, the median values for functions F3, F4, F6, F7, and F9 are located near the lower boundaries of the boxes, indicating a right-skewed (positively skewed) distribution. This suggests that the majority of runs yielded relatively favorable results. Moreover, no outliers are observed in functions F1, F4, F5, F7, or F9 and only a few outliers appear in F2, F3, F6, F8, and F10, demonstrating that DQDCS exhibits stronger robustness compared to the other algorithms.

#### 4.1.4. CEC2019 Wilcoxon Rank-Sum Test

The Wilcoxon rank-sum test [32], a non-parametric statistical test, was employed to evaluate the significant differences between two algorithms. The objective was to verify whether there exists a significant difference between the DQDCS algorithm and the other ten comparison algorithms, thereby assessing the optimization performance of DQDCS. A *p*-value below 0.05 indicates the rejection of the null hypothesis, signifying a significant difference between the two algorithms. Table 3 presents the *p*-values obtained from the Wilcoxon rank-sum test conducted between DQDCS and the other ten representative comparison algorithms when solving the CEC2019 benchmark. As shown in Table 3, DQDCS outperforms the other comparison algorithms in solving the CEC2019 functions, with *p*-values between DQDCS and each of the comparison algorithms being lower than 0.05. The results unequivocally emphasize that DQDCS exhibits significant differences compared to the other algorithms in the majority of the functions, highlighting its distinct advantage in solution performance.

### 4.2. The CEC2022 Benchmark Functions Are Employed for Performance Evaluation

The CEC2022 benchmark functions are a standardized set of problems used to assess the performance of optimization algorithms in research and development. These test functions simulate various aspects of real-world optimization problems, including local minima, maxima, global optima, and a range of complexities such as nonlinearity and discontinuities. By validating algorithms on these diverse test sets, it ensures that the algorithms exhibit high robustness and stability when confronted with different challenging scenarios. This approach helps avoid the issue of algorithms performing exceptionally well in specific environments while failing in others, thereby enhancing the generality and reliability of the algorithm. For a comprehensive evaluation, the complex functions described in the CEC2022 test suite are used to assess the effectiveness of the DQDCS algorithm. The number of iterations is set to 500, and 100 independent tests are conducted for each benchmark function. The population size is consistently established at 200 individuals.

#### 4.2.1. CEC2022 Optimization Accuracy Analysis

To visually observe and compare the results of the CEC2022 benchmark functions, the following table presents the optimal values (best), mean values (mean), and standard deviations (Std) for each test function. In these tables, the mean value is used as the performance indicator, and the best mean values are underlined. These results are provided in Table 4 below.

From the statistical results in Table 4, it can be observed that for test functions F1 to F3, DQDCS achieved the best mean values and found the optimal solutions when compared to the other algorithms. Moreover, when searching for the optimal values of functions F1 to F3, DQDCS exhibited the lowest standard deviation among all algorithms.

For test functions F4 to F8, DQDCS found the optimal solutions for functions F5 to F8. It achieved the best mean values across all algorithms for functions F6, F7, and F8. Additionally, for function F4, DQDCS outperformed DCS in terms of the mean value, and DQDCS also achieved lower standard deviations than the other algorithms in functions F4, F5, and F7.

In the case of test functions F9 to F12, DQDCS found the optimal solutions for functions F11 and F12. For functions F9, F10, and F12, DQDCS achieved the best mean values across all algorithms. For function F11, DQDCS outperformed DCS in terms of the mean value. Furthermore, DQDCS exhibited the lowest standard deviation in functions F10 and F12, and its standard deviation was lower than that of DCS in functions F9 and F11.

#### 4.2.2. CEC2022 Convergence Curve Analysis

**Figure 5:**
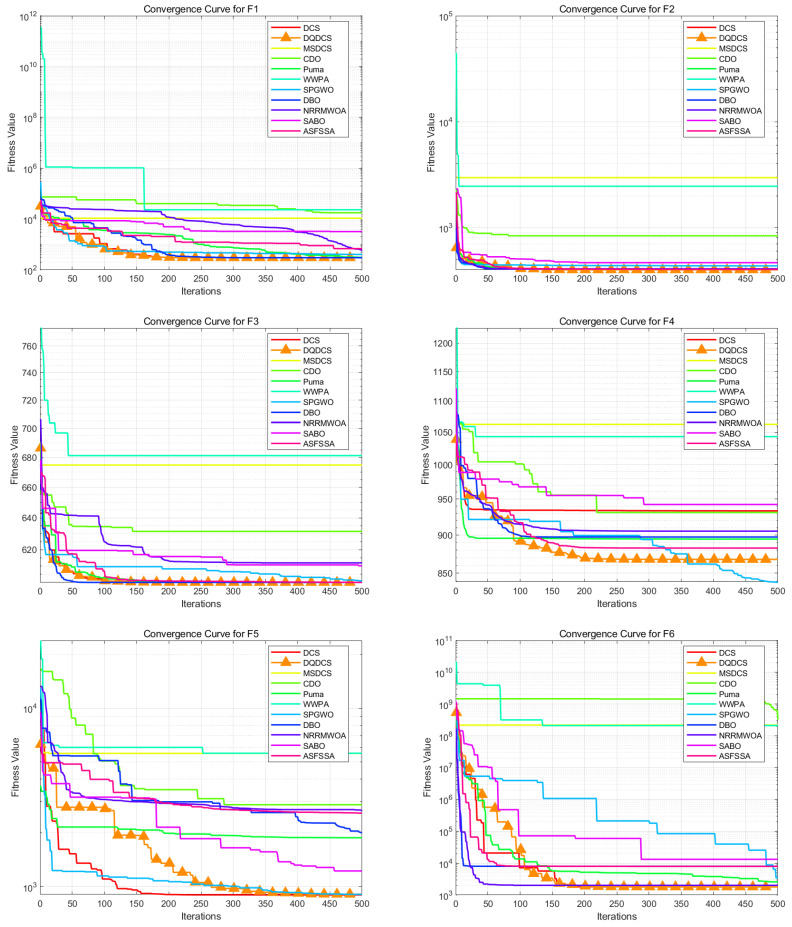

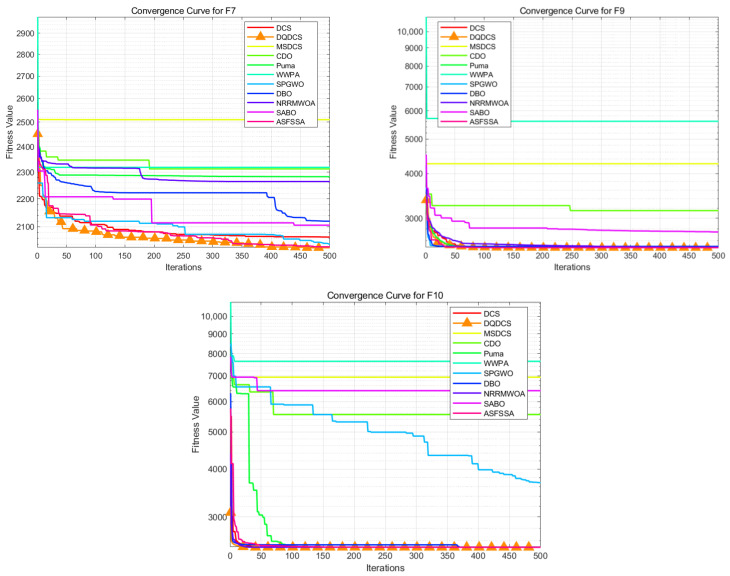
Convergence curve of CEC2022 benchmark tests.

To more intuitively compare the optimization accuracy and convergence speed of various algorithms, the convergence curves for each algorithm based on the CEC2022 benchmark functions are presented in Figure 5, which shows a comparison of the convergence curves for eleven algorithms, with the horizontal axis representing the number of iterations and the vertical axis representing fitness values. The DQDCS algorithm exhibits the highest convergence speed on functions F1, F4, F7, F8, F10, F11, and F12, achieving the highest convergence accuracy on functions F1 to F3 and F5 to F12. Furthermore, it almost finds the optimal value at the beginning, especially for functions F2, F8, F9, F10, and F12, where it converges to the optimal value in an almost linear manner. This further confirms that the DQDCS algorithm performs relatively well, and the multi-strategy improvements are effective and feasible in enhancing both the convergence speed and accuracy of the algorithm.

#### 4.2.3. CEC2022 Boxplot Analysis

**Figure 6:**
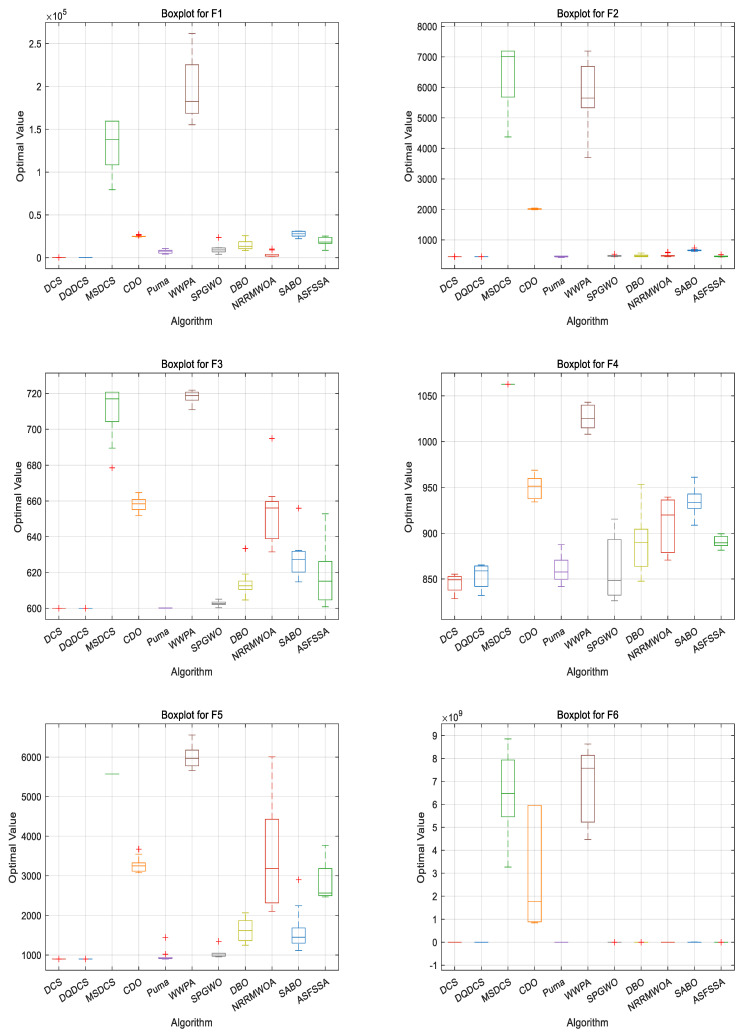

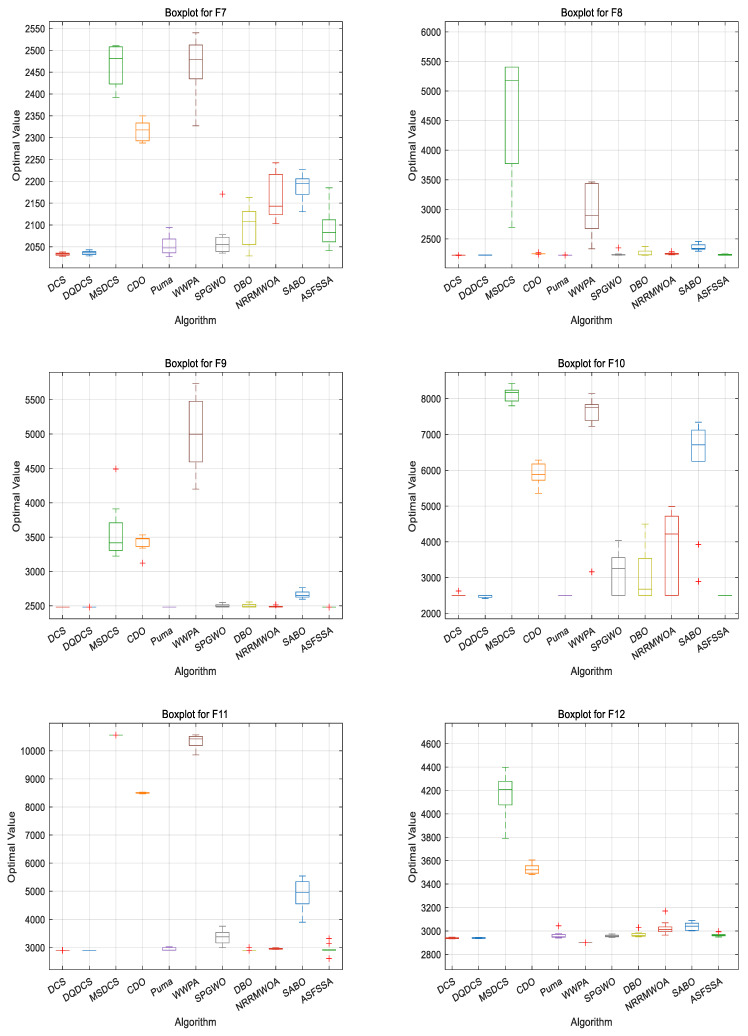
Boxplots of CEC2022 benchmark functions.

In order to compare the performance of different algorithms, a boxplot evaluation based on the CEC2022 benchmark functions was drawn to assess and compare the algorithms. As shown in Figure 6, the DQDCS algorithm exhibits no outliers across functions F1 to F12, indicating superior robustness compared to the other algorithms. Furthermore, the boxplots for functions F1 to F3 and F5 to F12 are relatively flat, suggesting that the DQDCS algorithm demonstrates stable data behavior.

#### 4.2.4. CEC2022 Wilcoxon Rank-Sum Test

To further evaluate the effectiveness of the DQDCS algorithm, the Wilcoxon rank-sum test was utilized. This test is ideal for comparing the performance of original and improved algorithms, as it can assess significant differences between two independent samples, particularly when the data do not follow a normal distribution. A key advantage of the Wilcoxon rank-sum test is its non-parametric nature, which makes it particularly effective for comparing two independent sets of samples without assuming a specific distribution, regardless of sample size.

By conducting the Wilcoxon rank-sum test on the DQDCS algorithm and other algorithms based on the CEC2022 test set, the superiority and reliability of the DQDCS algorithm were further evaluated. The specific results are shown in Table 5. The *p*-value indicates the degree of significance between the two algorithms. When the *p*-value is less than 5%, the difference is considered significant; otherwise, it is not. The results presented in Table 5 indicate that the *p*-values are all less than 5%, which demonstrates a significant difference between DQDCS and the other comparison algorithms, further validating the superiority and effectiveness of the DQDCS algorithm.

In conclusion, the DQDCS algorithm significantly enhances the overall performance of the DCS algorithm. When compared with other algorithms, it exhibits outstanding performance and strong overall capability. However, there are still some limitations to the DQDCS algorithm. For instance, the increased diversity in the initialization stage sacrifices some of the algorithm’s convergence speed. Additionally, when solving certain functions, the increased computational load leads to slight performance degradation. Therefore, there remains room for improvement. These results suggest that the hybrid multi-strategy approach is effective for most of the test functions, which aligns with the “no free lunch” theorem.

## 5. Engineering Case Studies and Results Analysis

In the research and development of metaheuristic optimization algorithms, the construction and refinement of algorithm performance evaluation systems have always been a central focus in the academic community. Traditional evaluation paradigms are often based on benchmark test function sets. While these functions provide a standardized testing environment and clear theoretical optimal solutions, they significantly differ from the complexities of real-world engineering problems, making it difficult to fully reflect the practical applicability of algorithms in real-world scenarios. In contrast, real-world engineering problems typically exhibit highly complex characteristics. First, the optimal global solution is difficult to determine in advance using analytical methods, and its existence and uniqueness often lack rigorous mathematical proof. Second, the problem space generally contains various complex constraints, such as nonlinear constraints, inequality constraints, and boundary conditions, which are interwoven and greatly increase the difficulty of solving the problem. These features make real-world engineering problems an ideal benchmark for testing the robustness, adaptability, and engineering practicality of optimization algorithms.

From both the algorithm validation and engineering application perspectives, employing real-world problems with actual engineering constraints as test cases holds irreplaceable significance for thoroughly evaluating the practical performance of optimization algorithms. This testing approach not only more accurately simulates the algorithm’s performance in real operational environments, but also effectively addresses the limitations of benchmark function tests in reflecting the generalization capability of algorithms. Based on this, this study carefully selected the design of static pressure thrust bearings [33] and the application of synchronous optimal pulse width modulation (SOPWM) in three-level inverters [34] as typical test cases. This study aimed to conduct a systematic empirical analysis to evaluate the effectiveness and superiority of the proposed algorithm in solving complex engineering optimization problems.

The design of static pressure thrust bearings is a typical multidisciplinary optimization problem involving fields such as fluid mechanics, materials science, and thermodynamics. The optimization of its design parameters requires balancing multiple performance metrics, including load-bearing capacity, stability, and energy consumption. On the other hand, the application of synchronous optimal pulse width modulation (SOPWM) in three-level inverters focuses on the field of power electronics, aiming to find the optimal solution among several objectives, such as ensuring output voltage waveform quality, reducing switching losses, and suppressing harmonics. These two engineering cases are highly representative and complementary: on one hand, they encompass various constraints from different engineering fields, such as mechanical and power electronics, which allows for a comprehensive assessment of the algorithm’s capability in handling complex constrained optimization problems; on the other hand, through in-depth analysis of real engineering data, the performance of the algorithm in terms of solution quality, efficiency, and convergence speed can be directly evaluated. The research results not only provide essential empirical evidence for further optimization of the algorithm but also lay a solid theoretical and practical foundation for its broader application in various engineering domains.

### 5.1. Static Pressure Thrust Bearing

The primary objective of this design problem is to optimize the bearing power loss using four design variables, with the goal of minimizing the power loss. These design variables include the bearing radius $R\left(x_{1}\right)$, groove radius $R_{0}\left(x_{2}\right)$, oil viscosity $\mu \left(x_{3}\right)$, and flow rate $Q\left(x_{4}\right)$. The problem involves seven nonlinear constraints, labeled $g_{1}–g_{7}$, which are defined in Equations (23)–(29). These constraints pertain to the load-carrying capacity *W*, the inlet oil pressure $P_{0}$, and the oil film thickness *h*, as specified in Equations (30), (31), and (32), respectively.

**Figure 7:**
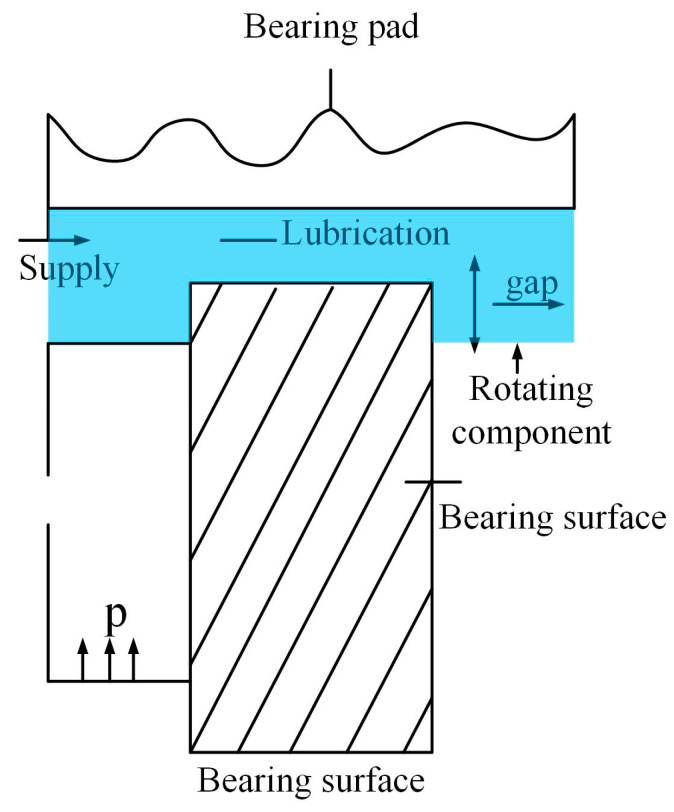
Static pressure thrust bearing. Arrows indicate the flow direction or movement direction.

The objective function f(x) primarily includes the flow rate of the lubricant, inlet oil pressure, and the power loss function resulting from friction under specific constraints. The detailed formulation is provided in Equation (22). Additionally, the power loss caused by friction is closely related to the temperature rise of the lubricant and the oil film thickness. The axial load constraint *W* ensures that the bearing can withstand the specified axial load, which is a fundamental requirement for its proper operation. This constraint is directly related to the bearing’s load-carrying capacity and stability. The inlet oil pressure constraint $P_{0}$ guarantees a reasonable inlet pressure, which is crucial for effective lubrication and normal operation of the bearing. Insufficient oil pressure may result in inadequate lubrication, while excessive pressure could lead to leakage or other failures. The oil film thickness constraint *h* ensures an appropriate film thickness, which is essential for reducing friction, minimizing power loss, and preventing surface wear. This constraint plays a critical role in maintaining the bearing’s performance and extending its service life. Figure 7 illustrates the structure of the static pressure thrust bearing, the blue background indicates regional visualization.

(22) $$f(x)=\frac{QP_{0}}{0.7}+E_{f}$$

In the objective function f(x): Q represents the flow rate of the lubricant oil; $P_{0}$ denotes the inlet oil pressure; and $E_{f}$ represents the power loss caused by friction. The constraints associated with the objective function are mainly composed of the following seven inequality constraints.

(23) $$g_{1}(x)=W-10,1000\leq 0$$

(24) $$g_{2}(x)=5000-\frac{W}{\pi (R^{2}-R_{0}^{2})}\leq 0$$

(25) $$g_{3}(x)=50-P_{0}\leq 0$$

(26) $$g_{4}(x)=0.001-\frac{0.0307}{386.4P_{0}}(\frac{Q}{2\pi Rh})\leq 0$$

(27) $$g_{5}(x)=R-R_{0}\leq 0$$

(28) $$g_{6}(x)=h-0.001\leq 0$$

(29) $$g_{7}(x)=h-0.001\leq 0$$

(30) $$W=\frac{\pi P_{0}}{2}\frac{R^{2}-R_{0}^{2}}{\ln(\frac{R}{R_{0}})}$$

(31) $$P_{0}=\frac{6\mu Q}{\pi h^{3}}\ln(\frac{R}{R_{0}})$$

The temperature rise expression can be calculated using Equations (32) and (33).

(32) $$\Delta T=2(10^{P}-559.7)$$

(33) $$P=\frac{\log_{10}\log_{10}(8.122\times 10^{6}\mu +0.8)+3.55}{10.04}$$

The frictional power loss, $E_{f}$, is given by Equation (34).

(34) $$E_{f}=9336Q\times 0.0307\times 0.5\Delta T$$

The film thickness, h, is defined as shown in Equation (35).

(35) $$h={\left(\frac{2\pi \times 750}{60}\right)}^{2}\frac{2\pi \mu }{E_{f}}(\frac{R^{4}}{4}-\frac{R_{0}^{4}}{4})$$

The remaining parameters are defined as shown in Equations (36)–(39).

(36) 1≤R≤16

(37) $$1\leq R_{0}\leq 16$$

(38) $$1\times 10^{-6}\leq \mu \leq 16\times 10^{-6}$$

(39) 1≤Q≤16

To compare the performance of DQDCS with several classical algorithms, the population size was set to 30, the number of iterations to 200, and each algorithm was executed 30 times. The results of the static pressure thrust bearing design problem are presented in Table 6, where the best values are underlined for clarity. It can be observed that DQDCS ranks first in terms of best solution, variance, mean, and worst-case performance. Additionally, the median value of DQDCS ties for first place with DBO. In summary, DQDCS demonstrates superior overall performance in solving this engineering problem.

### 5.2. Application of SOPWM (Synchronous Optimal Pulse Width Modulation) in Three-Level Inverter

**Figure 8:**
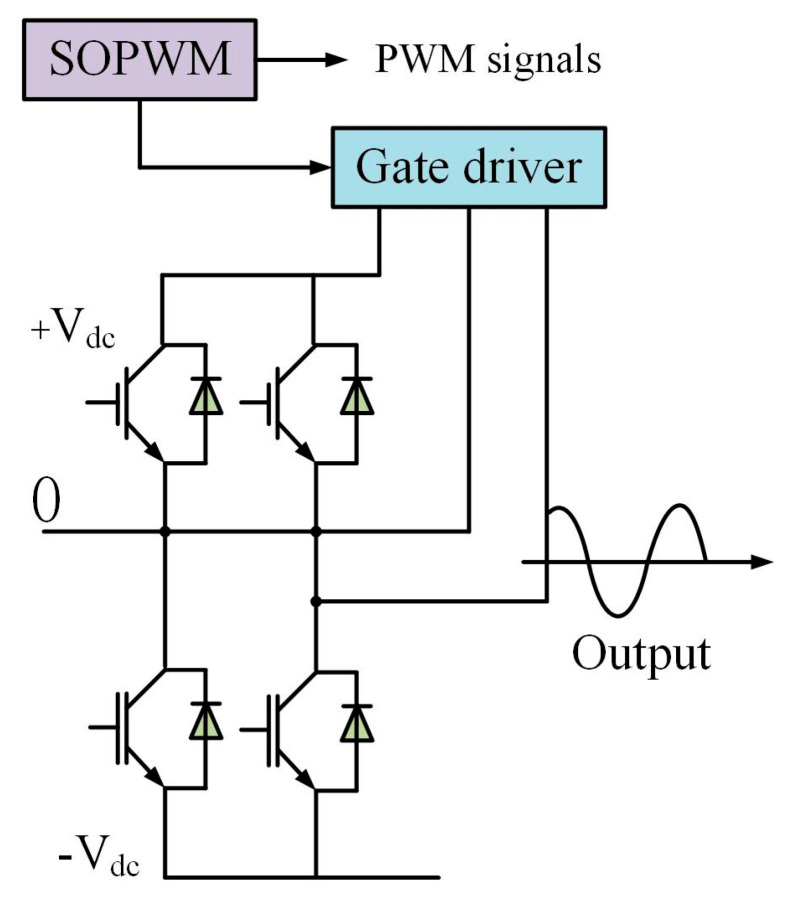
SOPWM for 3-level inverters. The arrow part represents the signal flow direction in the SOPWM schematic diagram.

Synchronous optimal pulse width modulation (SOPWM) is an advanced technique used for controlling medium-voltage (MV) drives. It significantly reduces the switching frequency without introducing additional distortion, thereby decreasing switching losses and improving the performance of inverters. Within one fundamental period, the switching angles are computed to minimize current distortion simultaneously. SOPWM can be transformed into a scalable constrained optimization problem. For inverters with different voltage levels, the application of SOPWM in three-level inverters can be described as follows. The primary objective of this problem is to minimize the current distortion *f*, subject to the constraints g and *h*, as described by Equations (40)–(43). Figure 8 illustrates the structure of the SOPWM application in a three-level inverter.

(40) $$f=\frac{\sqrt{\sum_{k}\left(k^{-4}\right){\left(\sum_{i=1}^{N}s\left(i\right)\cos\left(k\alpha_{i}\right)\right)}^{2}}}{\sqrt{\sum_{k}k^{-4}}}$$

(41) k=5,7,11,13,…,97,

(42) $$N=\left\lfloor \frac{f_{s,\max}}{f_{.m}}\right\rfloor$$

(43) $$s\left(i\right)={\left(-1\right)}^{i+1}$$

The constraint on the relationship between adjacent switching angles is defined by Equation (44). Specifically, it requires that the difference between two consecutive switching angles must exceed a threshold $10^{-5}$. This is intended to ensure a certain degree of regularity and stability in the variation of switching angles, thereby preventing potential control issues caused by excessively close switching angles. The constraint condition h(i) involves a modulation-related parameter m, and is expressed by Equation (45). This condition states that under specific circumstances, the sum of cosine terms involving $s\left(i\right)$ and α(i) must be equal to m. It serves to constrain the switching angles and other related parameters to ensure compliance with the operational requirements of the system. Equation (46) defines the constraint on the switching angle $\alpha_{i}$, restricting its values to a range between 0 and $\frac{\pi }{2}$. This limitation is established based on the physical characteristics and operational requirements of the inverter, ensuring that the switching angle varies within a reasonable range. Such a constraint is essential for maintaining proper inverter functionality and achieving optimal performance.

The proposed improved algorithm is compared against several classical algorithms using a population size of 30 and 200 iterations, with each algorithm executed 30 times. The experimental results for the application of synchronous optimal pulse width modulation (SOPWM) in a three-level inverter are presented in Table 7, where the best values are underlined for clarity. As observed, DQDCS achieves the best rank in terms of the optimal value and standard deviation, ranks second in both mean and median values, and ranks fourth in the worst-case performance. Taken together, these results demonstrate that DQDCS exhibits the best overall performance in solving this problem.

(44) $$g(i)=\alpha_{i+1}-\alpha_{i}-10^{-5}>0,i=1,2,\ldots ,N-1$$

(45) $$h(i)=m-\sum_{i=1}^{N}s\left(i\right)\cos\left(\alpha_{i}\right)=0$$

(46) $$0<\alpha_{i}<\frac{\pi }{2},i=1,2,\ldots ,N$$

### 5.3. Analysis of CPU Running Time for Each Algorithm

The CPU running time of an algorithm is a critical performance metric, directly affecting both the efficiency and responsiveness of a program. By analyzing time complexity, we can quantify the algorithm’s running time under various input sizes. Time complexity represents the number of CPU cycles required for the algorithm’s execution, assisting in predicting performance in large-data environments. The CPU running time is influenced not only by the algorithm itself but also by factors such as hardware architecture, compiler optimizations, and the operating environment. Practical testing and performance benchmarking enable the validation of theoretical analysis and provide a foundation for algorithm optimization.

**Figure 9:**
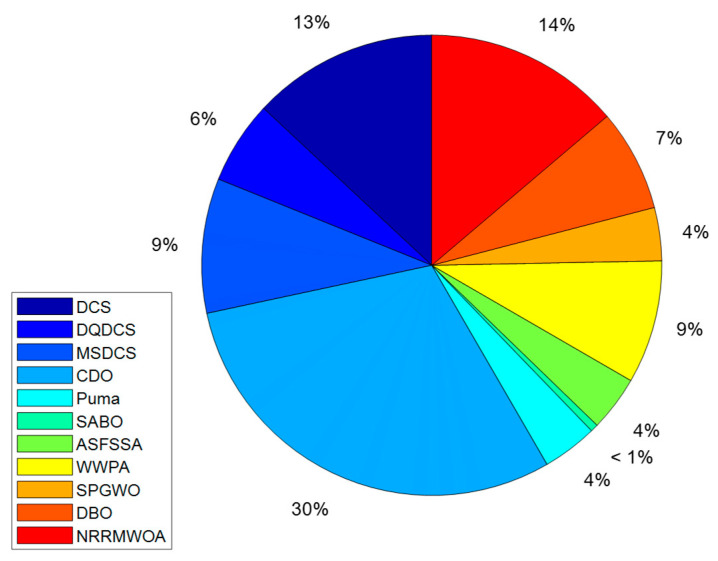
Comparison charts of CPU running times of various algorithms for static pressure thrust bearing.

Therefore, a comprehensive analysis of CPU running time serves as an essential tool for algorithm selection and system design. By comparing the CPU running times of different algorithms under identical conditions, the most efficient solution can be identified. As shown in Figure 9, the DQDCS algorithm ranks third in CPU running time when applied to the static pressure bearing problem. It significantly outperforms the original DCS algorithm, with shorter running times reducing hardware load, extending equipment lifespan, and lowering operational costs.

**Figure 10:**
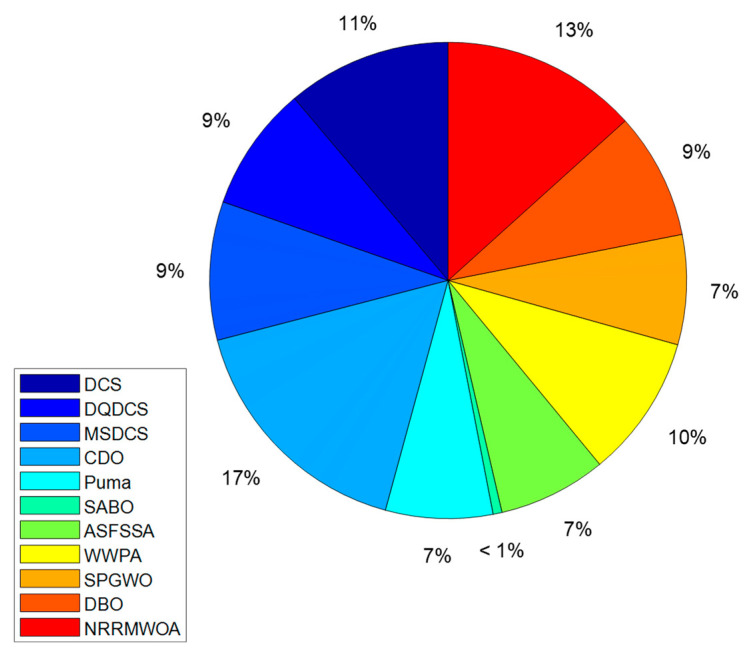
Comparison charts of CPU running times of various algorithms for SOPWM for 3-level inverters.

Moreover, the DQDCS algorithm also ranks third in CPU running time for solving the SOPWM (synchronous optimal pulse width modulation) problem in a three-level inverter, as depicted in Figure 10. The reduced CPU running time contributes to lower energy consumption, particularly in large-scale computations, resulting in savings in both electricity and hardware resources.

In summary, the DQDCS algorithm demonstrates excellent performance when addressing specific engineering problems, with its shorter CPU running time bringing significant economic and environmental benefits to real-world applications. These results further validate the importance of algorithm optimization and provide valuable insights for future algorithm design and enhancement. Through an in-depth analysis and comparison of CPU running times, we can select the most suitable algorithm for various computationally intensive tasks, thereby ensuring optimal resource utilization while maintaining computational efficiency.

## 6. Conclusions

A novel variant of the differential creative search (DCS) algorithm, termed DQDCS, is proposed to address the issue of uneven optimization performance in engineering applications. This improved version integrates a refined set-based clustering process and a double Q-learning mechanism. By leveraging a uniformly distributed initial population derived from the refined set and clustering process, the algorithm significantly reduces the risk of premature convergence to local optima in the early search phase, introducing a diverse set of individuals characterized by high randomness and non-determinism. The double Q-learning strategy is employed to effectively balance exploration and exploitation, enhancing the algorithm’s ability to escape local optima and substantially improving search efficiency and convergence accuracy.

Comparative simulation experiments were conducted between DQDCS, the original DCS, and several other state-of-the-art algorithms using both standard benchmark functions and constrained engineering optimization problems. The results demonstrate that DQDCS offers superior optimization speed and accuracy, maintaining its ability to avoid local optima even in later stages of the search. Specifically, DQDCS achieved 19 first-place rankings across the CEC2019 and CEC2022 benchmark functions, indicating robust and consistent performance. Furthermore, in the static thrust bearing design problem and the SOPWM (synchronous optimal pulse width modulation) application in three-level inverters, DQDCS consistently ranked first in overall performance, validating its effectiveness for solving real-world engineering optimization tasks.

The DQDCS algorithm involves substantial computational processes during each iteration, including population initialization, fitness evaluation, and position updating. These procedures can lead to extended computational times and increased demands on hardware resources, particularly when addressing large-scale or complex optimization problems. To enhance the efficiency of the algorithm, parallel computing strategies can be adopted. In this approach, individuals within the population are distributed across multiple processors or computational nodes, enabling concurrent execution and significantly accelerating the algorithm’s performance. While the DQDCS algorithm is primarily designed for continuous optimization problems, its application to discrete or combinatorial optimization tasks requires specific modifications. For discrete optimization problems, a feasible solution is to encode discrete variables into a continuous representation, execute the optimization within the continuous domain, and subsequently decode the solutions back to their original discrete forms. Furthermore, it is crucial to investigate and develop mutation, crossover, and other evolutionary operators explicitly adapted for discrete search spaces. Such enhancements are expected to improve the algorithm’s capability and efficiency in addressing discrete optimization challenges.

Future research will prioritize extending the application of the DQDCS algorithm to electro-hydraulic servo control and autonomous robotic arm control. Particular emphasis will be placed on the deep integration of reinforcement learning with DQDCS to further enhance its optimization capabilities. Additionally, future studies will explore the development of hybrid metaheuristic algorithms that incorporate multiple strategies or novel mathematical concepts. These improvements aim to enhance population diversity, maintain a balanced exploration-exploitation trade-off, and mitigate premature convergence, thereby improving optimization accuracy and accelerating convergence speed.

The DQDCS algorithm exhibits substantial potential in various engineering design and manufacturing fields. It can be effectively applied to mechanical structure optimization, enhancing strength, stiffness, and fatigue life while simultaneously reducing weight and minimizing costs. In the energy and power sectors, DQDCS can be employed to optimize power system generation scheduling and energy storage configurations, thereby improving overall efficiency and cost-effectiveness. Beyond these applications, DQDCS demonstrates promise in autonomous driving and robotics, where it can facilitate real-time path planning and trajectory optimization, ultimately enhancing safety and operational efficiency.

Despite its promising applications, DQDCS still faces certain limitations, such as computational inefficiencies in high-dimensional problems and local convergence challenges in complex constrained optimization tasks. To address these issues, future work will focus on refining the algorithm’s structure and parameter settings to enhance its solution efficiency and accuracy in high-dimensional, complex scenarios. Additionally, research will investigate more effective constraint-handling mechanisms to improve DQDCS’s global convergence ability and robustness in solving complex constrained optimization problems. These advancements aim to elevate the algorithm’s performance, making it more adaptable and resilient in real-world engineering applications.

## Figures and Tables

**Table 1:** Ablation experiment results for each strategy.

	Algorithm	Best	Mean	Std
F2	DCS	3.4322	33.4401	48.401
	D1	3.1725	10.5595	18.8376
	D2	3.1723	15.7383	19.5717
	D3	3.1523	15.7383	19.5717
	D4	3.1517	32.8329	49.4357
F5	DCS	1.0297	1.0793	0.047848
	D1	1.0357	1.1997	0.1064
	D2	1.0365	1.2023	0.1112
	D3	1.0198	1.2019	0.103
	D4	1.0008	1.0577	0.034179
F8	DCS	3.0423	3.5322	0.56543
	D1	2.8924	4.45	0.55146
	D2	2.3974	2.8518	0.3392
	D3	2.0233	2.8758	0.40339
	D4	2.0129	2.8364	0.3775
F10	DCS	7.7178	20.0627	3.5227
	D1	6.9765	18.3128	5.2696
	D2	5.3547	19.6528	4.7076
	D3	2.7937	19.535	5.1848
	D4	1.0001	19.9929	4.4706

**Table 2:** Experimental results on the CEC2019 benchmark functions. The underlines are used to highlight the optimal average values.

Function	Algorithm	Best	Mean	Std
F1	DCS	1	1.1746	0.066539
	DQDCS	1	1.0129	0.017456
	MSDCS	1.0001	54.4005	138.3435
	CDO	1	1.2	0
	Puma	1	103,218.6127	227,810.8443
	WWPA	1.6834	238.3049	583.4071
	SPGWO	1	3656.3391	8551.4486
	DBO	1	414,533.6708	721,175.8267
	NRRMWOA	180.8782	785,017.9378	1,311,767.1446
	SABO	1	5.7741	21.3505
	ASFSSA	1	1	0
F2	DCS	3.4322	33.4401	48.401
	DQDCS	3.1517	32.8329	49.4357
	MSDCS	5.1132	9.6797	4.1544
	CDO	5	5	0
	Puma	4.2328	4.7036	0.37262
	WWPA	5.2001	8.3147	3.6477
	SPGWO	63.7169	259.0299	142.4842
	DBO	4.2752	384.2964	194.8378
	NRRMWOA	11.9124	721.1422	965.2022
	SABO	4.5993	8.7857	5.9168
	ASFSSA	4.2189	4.3289	0.18135
F3	DCS	2.2424	2.9371	0.4211
	DQDCS	1.0004	2.0439	0.2884
	MSDCS	11.7269	12.4015	0.34845
	CDO	4.7096	5.8612	0.64681
	Puma	1.4656	2.1404	0.57859
	WWPA	8.0688	10.1437	0.86708
	SPGWO	2.4504	3.2425	0.74601
	DBO	1.4091	3.9766	1.8562
	NRRMWOA	1.4106	4.9893	1.953
	SABO	5.7387	6.9026	0.57406
	ASFSSA	1.0134	3.6414	1.5732
F4	DCS	5.1848	7.2552	1.0675
	DQDCS	5.01	5.5951	1.8712
	MSDCS	85.6623	136.8029	21.366
	CDO	63.5432	71.4357	5.479
	Puma	5.9748	13.0409	5.7055
	WWPA	116.9494	142.2562	13.0633
	SPGWO	4.0021	14.8937	9.5953
	DBO	11.0965	23.7472	8.109
	NRRMWOA	17.0311	43.543	18.719
	SABO	35.1635	45.1917	7.9818
	ASFSSA	7.9865	41.071	29.6237
F5	DCS	1.0297	1.0793	0.047848
	DQDCS	1.0008	1.0577	0.034179
	MSDCS	76.1748	153.0113	42.5413
	CDO	53.7219	72.8935	4.7508
	Puma	1.0271	1.1604	0.096242
	WWPA	122.0759	173.3345	21.0507
	SPGWO	1.1723	1.5255	0.22175
	DBO	1.0442	1.1462	0.06739
	NRRMWOA	1.2785	1.5229	0.17431
	SABO	1.7572	2.9426	0.93688
	ASFSSA	1.0615	1.1646	0.072103
F6	DCS	1.0004	1.9302	1.0947
	DQDCS	1.0029	1.4685	0.73351
	MSDCS	9.6249	14.1852	1.4021
	CDO	7.8188	9.4417	0.90669
	Puma	1.004	1.7517	0.89464
	WWPA	11.2646	12.8644	0.87031
	SPGWO	1.281	2.1444	0.92531
	DBO	2.0546	4.6142	1.7611
	NRRMWOA	4.9513	7.5365	1.5727
	SABO	2.8428	4.748	0.97675
	ASFSSA	1.0009	2.6429	1.1644
F7	DCS	119.6257	591.2146	214.4368
	DQDCS	80.447	347.2542	160.8203
	MSDCS	1910.947	2563.8317	271.9363
	CDO	1174.9817	1493.4973	180.7765
	Puma	126.3932	605.3863	286.0483
	WWPA	2117.4231	2407.1589	144.384
	SPGWO	342.6493	736.2504	217.0422
	DBO	417.6764	786.1656	309.1151
	NRRMWOA	499.5293	1150.2235	299.2043
	SABO	1214.0837	1760.7036	189.8127
	ASFSSA	293.3793	800.6022	282.4334
F8	DCS	3.0423	3.5322	0.56543
	DQDCS	2.447	3.365	0.022699
	MSDCS	4.654	5.2571	0.0764
	CDO	3.757	4.2063	0.20053
	Puma	2.4307	3.6169	0.41838
	WWPA	4.9553	5.2467	0.10746
	SPGWO	1.3278	3.4054	0.52501
	DBO	2.9084	3.8775	0.4717
	NRRMWOA	3.5071	4.3747	0.35839
	SABO	3.8848	4.5332	0.23968
	ASFSSA	3.1381	4.0752	0.33229
F9	DCS	1.0463	1.1288	0.052384
	DQDCS	1.0067	1.0426	0.040301
	MSDCS	3.8863	5.3393	0.61068
	CDO	3.6548	4.1862	0.14797
	Puma	1.0823	1.1672	0.051977
	WWPA	4.4026	5.2377	0.37346
	SPGWO	1.073	1.1543	0.040058
	DBO	1.1722	1.2769	0.08308
	NRRMWOA	1.1824	1.3798	0.15444
	SABO	1.1224	1.2898	0.066172
	ASFSSA	1.0916	1.1801	0.075728
F10	DCS	1.0044	20.2372	4.5283
	DQDCS	1.0001	19.9929	4.4706
	MSDCS	21.6494	21.6499	0.0010212
	CDO	21.2731	21.4023	0.061844
	Puma	20.9615	21.0133	0.027353
	WWPA	21.2641	21.6966	0.13883
	SPGWO	21.2315	21.3923	0.081047
	DBO	21	21.2387	0.18582
	NRRMWOA	20.996	21.0539	0.086024
	SABO	20.8947	21.3333	0.15323

**Table 3:** Wilcoxon rank-sum test results for CEC2019 benchmark.

DQDCS vs.	DCS	MSDCS	CDO	Puma	WWPA	SPGWO	DBO	NRRMWOA	SABO	ASFSSA
F1	6.39 × 10^−5^	1.83 × 10^−4^	1.82 × 10^−4^	1.72 × 10^−4^	1.83 × 10^−4^	1.13 × 10^−2^	4.45 × 10^−2^	1.83 × 10^−4^	1.83 × 10^−4^	6.35 × 10^−5^
F2	6.39 × 10^−5^	3.30 × 10^−4^	1.83 × 10^−4^	1.83 × 10^−4^	1.83 × 10^−4^	1.40 × 10^−1^	6.40 × 10^−2^	1.83 × 10^−4^	2.46 × 10^−4^	8.75 × 10^−5^
F3	2.11 × 10^−2^	1.83 × 10^−4^	7.30 × 10^−3^	5.83 × 10^−4^	1.83 × 10^−4^	2.80 × 10^−3^	2.57 × 10^−2^	5.83 × 10^−4^	5.80 × 10^−3^	1.73 × 10^−2^
F4	1.13 × 10^−2^	1.83 × 10^−4^	1.83 × 10^−4^	2.12 × 10^−2^	1.83 × 10^−4^	2.11 × 10^−2^	4.31 × 10^−1^	2.20 × 10^−3^	1.83 × 10^−4^	1.01 × 10^−3^
F5	6.40 × 10^−3^	1.83 × 10^−4^	1.83 × 10^−4^	1.83 × 10^−4^	1.83 × 10^−4^	1.83 × 10^−4^	1.40 × 10^−2^	1.13 × 10^−2^	1.83 × 10^−4^	1.83 × 10^−4^
F6	1.83 × 10^−4^	4.40 × 10^−4^	1.83 × 10^−4^	1.83 × 10^−4^	7.69 × 10^−4^	1.83 × 10^−4^	1.73 × 10^−2^	2.20 × 10^−3^	1.83 × 10^−4^	1.83 × 10^−4^
F7	2.46 × 10^−4^	1.83 × 10^−4^	1.83 × 10^−4^	3.76 × 10^−2^	1.83 × 10^−4^	7.69 × 10^−4^	4.52 × 10^−2^	5.80 × 10^−3^	1.83 × 10^−4^	5.80 × 10^−3^
F8	1.73 × 10^−4^	1.83 × 10^−4^	3.76 × 10^−2^	4.52 × 10^−2^	1.82 × 10^−4^	1.40 × 10^−2^	4.40 × 10^−4^	2.57 × 10^−2^	1.83 × 10^−4^	7.69 × 10^−4^
F9	7.75 × 10^−3^	1.83 × 10^−4^	1.83 × 10^−4^	5.80 × 10^−3^	1.83 × 10^−4^	3.39 × 10^−2^	2.57 × 10^−2^	4.52 × 10^−2^	7.69 × 10^−4^	2.20 × 10^−3^
F10	2.43 × 10^−2^	1.83 × 10^−4^	1.83 × 10^−4^	1.71 × 10^−3^	1.83 × 10^−4^	1.83 × 10^−4^	2.83 × 10^−3^	4.59 × 10^−3^	4.40 × 10^−4^	7.76 × 10^−4^

**Table 4:** Testing results of CEC2022 benchmark functions. The underline is the average value of the optimal performance in the operation test function and is used to highlight the optimal average value.

Function	Algorithm	Best	Mean	Std
F1	DCS	300	301	3.8519 × 10^−13^
	DQDCS	300	300	6.8317 × 10^−14^
	MSDCS	10,766.7503	10,766.7571	0.013964
	CDO	7490.1964	20,512.7673	13,607.6136
	Puma	300.0023	301.2711	2.5835
	WWPA	8455.8816	236,150.7116	1,491,237.9285
	SPGWO	304.8303	797.5061	1028.1107
	DBO	300	361.9946	341.4715
	NRRMWOA	358.8157	3032.3131	2230.1134
	SABO	1195.7814	3379.0063	1311.4349
	ASFSSA	309.4622	586.0842	281.7238
F2	DCS	400	405.5275	3.4253
	DQDCS	400.0035	403.425	3.6114
	MSDCS	750.1737	2491.5488	960.6972
	CDO	570.9415	835.2859	49.4592
	Puma	400	406.3943	7.2844
	WWPA	972.5671	3270.4214	1414.9336
	SPGWO	400.5352	415.7896	14.3696
	DBO	400.0218	424.2427	29.16
	NRRMWOA	400.0781	422.0518	27.9517
	SABO	404.4744	448.4682	21.9722
	ASFSSA	400.0102	413.4404	19.3205
F3	DCS	600	600	3.5703 × 10^−7^
	DQDCS	600.0001	600.0005	3.2297 × 10^−7^
	MSDCS	643.6113	682.7551	10.18
	CDO	627.5045	634.8725	3.539
	Puma	600	600	0.00026748
	WWPA	662.8221	687.3778	7.5308
	SPGWO	600.0541	600.4967	0.68095
	DBO	600	601.7739	2.6285
	NRRMWOA	601.6986	621.4008	11.8169
	SABO	603.078	612.0294	7.4192
	ASFSSA	600	602.2958	6.5591
F4	DCS	803.5611	809.1824	2.9573
	DQDCS	801.7438	809.0859	1.0729
	MSDCS	867.3885	896.995	4.0671
	CDO	828.5143	845.4771	6.2154
	Puma	806.9647	818.357	6.8807
	WWPA	864.4186	887.3275	8.2043
	SPGWO	800.3831	800.9999	6.6974
	DBO	807.9597	829.8866	10.517
	NRRMWOA	809.95	835.8238	14.4457
	SABO	819.7184	838.0276	8.1862
	ASFSSA	811.9395	829.6198	5.4414
F5	DCS	900	900	2.2852 × 10^−14^
	DQDCS	900	900	2.0549 × 10^−14^
	MSDCS	2066.3081	2246.8891	21.1599
	CDO	1248.3716	1377.5434	69.9206
	Puma	900	900.3861	0.57066
	WWPA	1860.8209	2380.0621	185.2868
	SPGWO	900	900.0042	10.5999
	DBO	900	916.4121	57.1606
	NRRMWOA	906.326	1253.0823	278.8837
	SABO	901.1913	925.9241	16.9796
	ASFSSA	901.7282	1398.8497	174.1701
F6	DCS	1800.0368	1805.6947	1.5327
	DQDCS	1800.0238	1801.939	2.0455
	MSDCS	70,252,227.0095	207,374,163.4593	33,963,697.1425
	CDO	15,167,928.0263	157,093,751.8526	240,080,753.4813
	Puma	1807.9235	2063.024	734.8686
	WWPA	8,608,986.8261	185,293,465.0815	73,329,210.9276
	SPGWO	1960.6464	5799.1861	2292.3454
	DBO	1895.1412	4731.5419	2375.8384
	NRRMWOA	1846.6869	3977.1499	2076.5281
	SABO	2459.4489	20,110.6228	11,920.7947
	ASFSSA	1930.1248	5432.4366	1903.6817
F7	DCS	2027.974	2049.2777	14.7551
	DQDCS	2000.229	2001.6385	2.837
	MSDCS	2337.8697	2447.3738	69.1571
	CDO	2231.4056	2300.052	30.0378
	Puma	2151.4621	2192.5272	43.688
	WWPA	2306.8139	2451.1073	90.2488
	SPGWO	2034.3498	2057.4032	25.2011
	DBO	2032.3433	2090.2749	34.7803
	NRRMWOA	2101.6025	2198.9024	60.3948
	SABO	2105.6682	2178.9918	37.0986
	ASFSSA	2030.9544	2094.5985	29.2648
F8	DCS	2222.0212	2225.7348	5.0729
	DQDCS	2200.7186	2208.8764	7.0158
	MSDCS	2846.8452	3952.5994	1101.3132
	CDO	2243.644	2251.946	6.6606
	Puma	2226.3281	2353.5188	125.6302
	WWPA	2551.5422	2917.5036	244.8562
	SPGWO	2224.4404	2229.9677	4.8594
	DBO	2233.7582	2301.6076	63.7515
	NRRMWOA	2237.5665	2262.526	34.2888
	SABO	2278.1633	2356.1119	64.2682
	ASFSSA	2222.1835	2227.1796	4.6573
F9	DCS	2529.2844	2529.2844	0
	DQDCS	2480.7821	2480.2942	0.027562
	MSDCS	3371.8015	3996.9709	412.0453
	CDO	3151.4652	3426.8386	126.0153
	Puma	2480.7976	2480.8202	0.017425
	WWPA	3370.7773	4492.3652	979.6033
	SPGWO	2481.1805	2500.7716	22.002
	DBO	2480.9125	2496.6431	20.4099
	NRRMWOA	2481.1486	2491.6469	14.7268
	SABO	2603.3814	2699.6239	46.6498
	ASFSSA	2480.7813	2480.8064	0.067892
F10	DCS	2500.3438	2515.1856	46.6588
	DQDCS	2500.1542	2503.504	18.4699
	MSDCS	6937.0014	7963.4014	476.9702
	CDO	4832.6414	5873.0597	506.4387
	Puma	2500.638	2515.0468	45.15
	WWPA	7228.1772	7552.068	210.5838
	SPGWO	2500.5073	3312.8457	742.2508
	DBO	2500.8157	2930.6677	678.0253
	NRRMWOA	2501.3194	4062.9987	1135.7437
	SABO	2858.03	5556.1544	1635.5186
	ASFSSA	2500.7384	2630.7374	410.1658
F11	DCS	2600	2646.5043	108.0761
	DQDCS	2600	2639	103.2576
	MSDCS	5105.1466	5105.1769	0.082861
	CDO	3329.1224	3343.2781	5.6324
	Puma	2600.0001	2637.3103	118.4636
	WWPA	3712.7414	4917.2808	262.7002
	SPGWO	2601.1884	2904.431	130.4169
	DBO	2600	2814.9987	171.69
	NRRMWOA	2600.698	2901.0184	130.9309
	SABO	2832.4972	3233.2798	104.4736
	ASFSSA	2600	2663.175	113.4852
F12	DCS	2988.2699	3219.0423	181.9443
	DQDCS	2700.6186	2722.0363	2.1318
	MSDCS	3624.3346	4070.0287	301.5474
	CDO	3478.4691	3508.6705	25.8794
	Puma	2939.2542	2951.6242	13.3077
	WWPA	2900.0048	2900.005	5.3612 × 10^−5^
	SPGWO	2937.2174	2955.9256	13.3377
	DBO	2939.8499	2973.7347	40.5269
	NRRMWOA	2958.0579	3044.9336	70.3033
	SABO	2994.7804	3054.3338	38.4792
	ASFSSA	2945.5917	2962.4681	13.8506

**Table 5:** Wilcoxon rank-sum test results for CEC2022 benchmark functions.

DQDCS vs.	DCS	MSDCS	CDO	Puma	WWPA	SPGWO	DBO	NRRMWOA	SABO	ASFSSA
F1	4.40 × 10^−4^	1.83 × 10^−4^	1.83 × 10^−4^	1.83 × 10^−4^	1.83 × 10^−4^	7.69 × 10^−4^	1.83 × 10^−4^	4.40 × 10^−4^	1.83 × 10^−4^	1.83 × 10^−4^
F2	3.61 × 10^−3^	1.83 × 10^−4^	1.83 × 10^−4^	1.83 × 10^−4^	1.83 × 10^−4^	1.83 × 10^−4^	8.90 × 10^−3^	1.83 × 10^−4^	1.83 × 10^−4^	1.83 × 10^−4^
F3	2.83 × 10^−3^	1.83 × 10^−4^	1.83 × 10^−4^	1.83 × 10^−4^	1.83 × 10^−4^	2.20 × 10^−3^	1.83 × 10^−4^	1.83 × 10^−4^	1.83 × 10^−4^	1.83 × 10^−4^
F4	9.11 × 10^−3^	1.83 × 10^−4^	1.83 × 10^−4^	4.52 × 10^−2^	1.83 × 10^−4^	1.13 × 10^−2^	2.20 × 10^−3^	8.90 × 10^−3^	1.83 × 10^−4^	5.21 × 10^−3^
F5	2.57 × 10^−2^	1.83 × 10^−4^	1.83 × 10^−4^	1.83 × 10^−4^	1.83 × 10^−4^	1.01 × 10^−3^	1.83 × 10^−4^	1.83 × 10^−4^	1.83 × 10^−4^	1.83 × 10^−4^
F6	6.23 × 10^−2^	1.83 × 10^−4^	1.83 × 10^−4^	1.83 × 10^−4^	1.83 × 10^−4^	1.73 × 10^−2^	1.40 × 10^−2^	2.20 × 10^−3^	1.83 × 10^−4^	4.40 × 10^−4^
F7	3.12 × 10^−2^	1.83 × 10^−4^	1.83 × 10^−4^	1.83 × 10^−4^	1.83 × 10^−4^	3.34 × 10^−2^	2.83 × 10^−3^	1.83 × 10^−4^	1.83 × 10^−4^	6.40 × 10^−2^
F8	3.12 × 10^−2^	1.83 × 10^−4^	1.83 × 10^−4^	4.40 × 10^−4^	1.83 × 10^−4^	2.20 × 10^−3^	1.04 × 10^−2^	1.83 × 10^−4^	1.83 × 10^−4^	4.40 × 10^−4^
F9	1.83 × 10^−4^	1.83 × 10^−4^	1.83 × 10^−4^	1.83 × 10^−4^	1.83 × 10^−4^	1.83 × 10^−4^	2.80 × 10^−3^	1.83 × 10^−4^	1.83 × 10^−4^	1.83 × 10^−4^
F10	1.71 × 10^−2^	1.83 × 10^−4^	1.83 × 10^−4^	2.21 × 10^−3^	1.83 × 10^−4^	1.73 × 10^−2^	9.11 × 10^−3^	1.83 × 10^−4^	1.83 × 10^−4^	3.09 × 10^−2^
F11	4.52 × 10^−2^	1.83 × 10^−4^	1.83 × 10^−4^	1.83 × 10^−4^	1.83 × 10^−4^	1.40 × 10^−2^	2.20 × 10^−3^	1.83 × 10^−4^	1.83 × 10^−4^	1.83 × 10^−4^
F12	1.73 × 10^−2^	1.83 × 10^−4^	1.83 × 10^−4^	1.01 × 10^−3^	1.83 × 10^−4^	7.69 × 10^−4^	1.01 × 10^−3^	2.46 × 10^−4^	1.83 × 10^−4^	2.46 × 10^−4^

**Table 6:** Optimization results of static pressure thrust bearing. The underline is the average value of the optimal performance in the operation test function and is used to highlight the optimal average value.

Name	*x*_1_	*x*_2_	*x*_3_	*x*_4_	Worst	Best	Std	Mean	Median
DCS	1.59727828 × 10^−5^	1.00023775	14.7915355	15.9942442	1.06923430 × 10^9^	1.07206086 × 10^9^	6.61825327 × 10^5^	1.07014876 × 10^9^	1.06997430 × 10^9^
DQDCS	1.60000000 × 10^−5^	1.00000000	14.8001689	16.0000000	1.06894753 × 10^9^	1.06954809 × 10^9^	1.34312464 × 10^5^	1.06898081 × 10^9^	1.06894753 × 10^9^
MSDCS	1.17291046 × 10^−5^	1.79130942	5.70274004	15.9413101	1.40136705 × 10^9^	5.22419187 × 10^14^	1.19485993 × 10^14^	3.57212782 × 10^13^	1.17985612 × 10^11^
Puma	1.60000000 × 10^−5^	1.00000000	14.8003474	16.0000000	1.06896145 × 10^9^	1.12825463 × 10^9^	1.37399596 × 10^7^	1.07613384 × 10^9^	1.07044585 × 10^9^
CDO	1.60000000 × 10^−5^	1.00000000	14.8163617	16.0000000	1.07047191 × 10^9^	1.15997503 × 10^9^	2.15231379 × 10^7^	1.09774625 × 10^9^	1.09665275 × 10^9^
WWPA	0.47339639	1.60123665 × 10^6^	3.94298347 × 10^5^	3.91671659 × 10^5^	−2.22716152 × 10^11^	2.35796666 × 10^10^	5.37550187 × 10^10^	1.52405939 × 10^10^	1.15527859 × 10^9^
SPGWO	1.60000000 × 10^−5^	1.00000000	14.8012444	1.60000000	1.06904448 × 10^6^	1.32319148 × 10^9^	5.65555937 × 10^7^	1.08297369 × 10^9^	1.07012621 × 10^9^
DBO	1.60000000 × 10^−5^	1.00000000	14.8001689	16.0000000	1.06894753 × 10^9^	1.47869424 × 10^9^	9.15951602 × 10^7^	1.08955220 × 10^9^	1.06894753 × 10^9^
NRRMWOA	1.60000000 × 10^−5^	1.00000000	14.8001572	16.0000000	1.06894764 × 10^9^	1.10966892 × 10^9^	1.11408274 × 10^7^	1.07630514 × 10^9^	1.07166285 × 10^9^
SABO	1.60000000 × 10^−5^	1.00000000	14.8145609	16.0000000	1.07029905 × 10^9^	1.49353781 × 10^9^	1.44018113 × 10^8^	1.18985405 × 10^9^	1.12582885 × 10^9^
ASFSSA	1.60000000 × 10^−5^	1.00000000	14.8002442	16.0000000	1.06895191 × 10^9^	1.07108086 × 10^9^	6.86835160 × 10^5^	1.06948901 × 10^9^	1.06929758 × 10^9^

**Table 7:** Optimization results of SOPWM for 3-level inverters. The underline is the average value of the optimal performance in the operation test function and is used to highlight the optimal average value.

Name	*x*_1_	*x*_2_	*x*_3_	*x*_4_	Worst	Best	Std	Mean	Median
DCS	0.556990820	1.55691929	1.55632264	1.57069023	0.995709896	1.00272898	1.67135890 × 10^−3^	0.998300957	0.998148422
DQDCS	0.556423139	1.57079633	1.57037521	1.57079633	0.995405132	0.995440460	7.97475504 × 10^−6^	0.995408550	0.995405849
MSDCS	0.398818649	1.41758251	1.49139957	1.54923355	1.61761236	105.741270	30.1338199	27.6842562	18.69082921
Puma	0.556414603	1.57079633	1.57037818	1.57079633	0.995405129	0.995580896	5.19507407 × 10^−5^	0.995430761	0.995406675
CDO	0.55583451001	1.57079633	1.57079633	1.57079633	0.995593045	1.14082188	6.15210384 × 10^−2^	1.05110186	1.00665276
WWPA	2.85068113 × 10^−3^	1.48395345 × 10^−4^	1.5715065 × 10^−4^	7.21160329 × 10^4^	1.48895835	78.0431264	18.2177239	11.9310968	4.09729542
SPGWO	0.556714311	1.57079633	1.57027734	1.57079633	0.995418349	1.16755946	6.26457711 × 10^−2^	1.02849798	0.995582535
DBO	0.556418317	1.57079633	1.57037750	1.57079633	0.995405127	1.16755679	3.97313485 × 10^−2^	1.00687810	0.995580896
NRRMWOA	0.556416113	1.57079633	1.57037874	1.57079633	0.995405128	1.16755772	5.29815832 × 10^−2^	1.01263761	0.995405148
SABO	0.555660648	1.57079633	1.57079633	1.57079633	0.995581236	0.996458735	2.43692395 × 10^−4^	0.995726761	0.995624926
ASFSSA	0.556418323	1.57079633	1.57037750	1.57079633	0.995405127	1.20665475	7.59187361 × 10^−2^	1.04151473	0.995508388

## Data Availability

The data used to support the findings of this study are available from the corresponding author upon request.
